# Overdominance Effect of the Bovine Ghrelin Receptor (*GHSR1a*)-*DelR242* Locus on Growth in Japanese Shorthorn Weaner Bulls: Heterozygote Advantage in Bull Selection and Molecular Mechanisms

**DOI:** 10.1534/g3.114.016105

**Published:** 2014-12-23

**Authors:** Masanori Komatsu, Yoichi Sato, Tatsuki Negami, Tohru Terada, Osamu Sasaki, Jumpei Yasuda, Aisaku Arakawa, Chikara Yoshida, Hideaki Takahashi, Aduli E. O. Malau-Aduli, Keiichi Suzuki, Kentaro Shimizu

**Affiliations:** * Animal Breeding Research Group, NARO Institute of Livestock and Grassland Science, National Agriculture and Food Research Organization (NARO), Tsukuba, Ibaraki 305-0901, Japan; † Department of Biotechnology, Graduate School of Agriculture and Life Sciences, The University of Tokyo, Tokyo 113-8657, Japan; ‡ Agricultural Bioinformatics Research Unit, Graduate School of Agriculture and Life Sciences, The University of Tokyo, Tokyo 113-8657, Japan; § Animal Industry Research Institute, Iwate Agricultural Research Center (IARC), Takizawa, Iwate 020-0173, Japan; ** Animal Science and Genetics, Tasmanian Institute of Agriculture, School of Land & Food, Faculty of Science, Engineering and Technology, University of Tasmania, Hobart, Tasmania TAS 7001, Australia; †† Veterinary and Biomedical Sciences, College of Public Health, Medical and Veterinary Sciences, James Cook University, Townsville, Queensland QLD 4811, Australia; ‡‡ Animal Research Group, National Institute of Agrobiological Sciences, Ikenodai 2, Tsukuba, Ibaraki 305-0901, Japan; §§ Graduate School of Agriculture, Tohoku University, Sendai, Miyagi 981-8555, Japan

**Keywords:** overdominance, growth, ghrelin receptor (GHSR1a), *DelR242*, cattle

## Abstract

Ghrelin and the ghrelin receptor (GHSR1a) are involved in growth hormone secretion, food intake, and several other important functions. Ghrelin acts on GHSR1a and induces signal transduction via the Gαq subunit. In our previous study, we identified the *DelR242* (*3R*) allele, a truncated 3-arginine residue (3R) [major type: 4 arginine residues (4R)] of the third intracellular loop of GHSR1a, with a high frequency in Japanese Shorthorn bulls (0.43) but with a low frequency in other cattle breeds (0.00–0.09). To further investigate the reasons for the higher frequency of the *3R* allele, we performed several experiments. In this study, we found a significant sex difference in the frequency of the *3R* allele. Statistical analysis revealed a significant overdominance effect of the *DelR242* locus on growth in Japanese Shorthorn weaner bulls. However, additive/dominance/overdominance effects of the *3R* allele on carcass traits in adult steers and dams were not significant. The mode of the overdominance effect was estimated to be solely controlled by the single *DelR242* locus without any other linked loci using linkage disequilibrium analysis in *GHSR1a*. These results indicated that *4R/3R* heterozygotes had a selective advantage in weaner bulls because of their higher average daily gain than homozygotes. We discussed possible molecular mechanisms involved in the overdominance effect of the *DelR242* locus on these traits in weaner bulls using a structural model of the complex consisting of a GHSR1a dimer and Gαq.

Japanese Shorthorn cattle were developed by crossbreeding indigenous (Nanbu-gyu) and Shorthorn (meat-type and/or dairy-type) cattle imported from the United States, England, Canada, and Australia in the Meiji to Showa eras in the northern Tohoku district (consisting of Iwate, Aomori, and Akita prefectures) and Hokkaido Island. Japanese Shorthorn cattle are superior in terms of hardiness in a cold climate and grazing ability; however, they show less uniformity in body weight (BW) and body conformation than Japanese Black cattle because of their high genetic diversity and unique crossbreeding histories. Natural mating on regional public ranches (standard herd: 1 sire and 50 dams) is a conventional practice (Mizuma and Sasaki 1974; Japanese Shorthorn Registry Association 1980).

The growth hormone secretagogue receptor 1a (GHSR1a), a ghrelin receptor, is a typical G-protein-coupled receptor (GPCR) with seven transmembrane domains, and *GHSR1a* mRNA is highly expressed in the hypothalamus and anterior pituitary gland (Howard *et al.* 1996). Both ghrelin, the endogenous ligand of GHSR1a, and GHSR1a itself are involved in growth hormone (GH) secretion, appetite regulation, taste bud signaling, and several other important functions (Sun *et al.* 2004; Kojima and Kangawa 2005; Cruz and Smith 2008; Shin *et al.* 2010; Albarran-Zeckler *et al.* 2011). In the study of nucleotide polymorphisms of bovine *GHSR1a*, we identified the *DelR242* (*3R*) allele, a truncated 3-arginine residue (3R) [major type: 4-arginine residue (4R)] of the third intracellular loop 3 (ICL3) of the GHSR1a protein and showed that it occurred with high frequency (0.43) in Japanese Shorthorn bulls and with low frequency (0.09) in Holstein–Friesian cattle. However, this allele was never found in Japanese Black or Mishima Island cattle (Komatsu *et al.* 2010). The 3R is a fundamental type of the GHSR1a protein in mice, rats, and Chinese hamsters, whereas the 4R is found in humans and pigs (Supporting Information, Figure S1). In humans, two missense mutations (p.R237W and p.D246A) adjacent to the 4R region of ICL3 of the GHSR1a protein have been reported in short-stature family lines (Pantel *et al.* 2009; Inoue *et al.* 2011). These GHSR1a mutations displayed a partial loss of constitutive activity of the GHSR1a receptor. We hypothesized that *DelR242* affects growth and carcass traits (Komatsu *et al.* 2010). We further investigated the causal trigger for the higher frequency of the *3R* allele of *GHSR1a* observed in Japanese Shorthorn cattle.

## Materials and Methods

### Animals and data collection

All livestock handling and cattle management procedures were performed in accordance with the guidelines for animal care of the NARO Institute of Livestock and Grassland Science. Genomic DNA was extracted from leukocytes using the standard phenol extraction protocol. Fragment analysis and sequencing of the *DelR242* locus, the 5′UTR microsatellite [*5*′*UTR-(TG)_n_*], the intron 1 microsatellite [*MS(GTTT)_5 > 6_*], and single-nucleotide polymorphisms (SNPs) of the *nt-7(C > A)*, *nt70(C > G)*, *nt456(G > A)*, *nt580(G > A)*, and *nt667(C > T)* loci were performed as previously described (Table S1) (Komatsu *et al.* 2010). The *nt70(C > G)*, *nt580(G > A)*, and *nt667(C > T)* loci in exon 1 and the intron 1 microsatellite [*MS(GTTT)_5 > 6_*] were fixed for the homozygotes [*C/C*, *G/G*, *C/C*, and *(GTTT)_5_/(GTTT)_5_*, respectively] in 95 sires. To compare allele and haplotype frequencies of the *DelR242*, *5′UTR-(TG)_n_*, *nt-7(C > A)*, and *nt456(G > A)* loci in sires and dams and to perform an association study between these loci and directly tested traits of weaner bulls or carcass traits of shipped half sibs, we collected a total of 661 genomic DNA samples [121 for sires and 540 for shipped half sibs sired by 17 bulls (210 dams and 330 steers)]. Of 121 weaner bulls, 26 individuals had only *DelR242* and *nt-7(C > A)* genotype data. Of 540 shipped half sibs, 3 individuals were excluded from analysis because of insufficient growth. The 540 half sibs were not randomly selected from the shipped steer and dam population because we planned to collect as many *3R/3R* individuals as possible for the association study using 540 half sibs sired by 14 *4R/3R* heterozygotes and 3 *3R/3R* homozygotes. In addition, the frequencies of the *3R* allele of the *DelR242* locus in 17 sires and 540 half sibs were significantly higher than those in 95 sires (Table S2). We estimated the allele or haplotype frequency in the dam population by removing the sire allele or haplotype from the 540 half sib genotypes or haplotype combinations.

Two experiments were performed for the association study. In Experiment 1 (Exp. 1), data of 121 weaner bulls in a direct-testing program in Iwate Prefecture from 1995 to 2009 (except for 1998) were analyzed. The calves were born between February and May in their respective farmers’ feedlots (birth years: 1995–2009) and grazed on regional public ranches with their dams from May to the end of September. Unfortunately, the birth weights of calves were not recorded. Preliminary selection on the basis of their body shape and conformation measurements (BSCMs) was performed at 4–5 months of age (Figure S3). The selected weaner bulls aged 6–7 months were transported to the direct testing station at the Animal Industry Research Institute, Iwate Agricultural Research Center (IARC). They were fed concentrate (6.3% of BW^0.75^/d, recalculated every 2 wk). They also had *ad libitum* access to roughage (timothy-grass hay) with an allowance of 42 d for adjustment and acclimatization, followed by the standard 140 d of direct testing prior to slaughter. The average age at the start of direct testing was 245.1 ± 20.6 d (mean ± SD). Routine management of the animals involved fortnightly recording of BW and BSCM traits at the start and end of direct testing [11 BSCM traits: withers height (WH); hip height (HH); body length (BL); chest depth (CD); chest width (CW); rump length (RL); hip width (HW); thurl width (TW); pin bone width (PBW); chest girth (CG); cannon circumference (CC)] (Figure S3). Other data included daily concentrate and roughage intakes, average daily gain (ADG) for the direct-testing period, total subcutaneous fat thickness at 8 points (8SFT), and total weight of concentrate and roughage intakes and concentrate and roughage weights per kg of BW gain (CONC_1 and ROUGH_1, respectively). Relative percentages (%) of roughage (ROUGH) were calculated from the daily recorded concentration and roughage intakes ([roughage]/[roughage + concentration]) during the direct-testing period. BWs observed at selection (BW_Sel) (average: 187 d of age), at the start (BW_S; average: 246 d of age), and at the end (BW_E; average: 386 d of age) of direct testing were used. The 180-d and 365-d adjusted BWs (180BW and 365BW, respectively) were estimated using simple linear regression with BW_Sel, BW_S, and BW_E as independent variables. Of 121 weaner bulls, 27 individuals (from 1995 to 2001) had data only for ADG, BW_S, BW_E, 180BW, 365BW, 8SFT, CONC_1, ROUGH_1, and ROUGH.

In Experiment 2 (Exp. 2), we used 537 half sibs (209 dams and 328 steers) sired by 17 bulls (the *DelR242* genotype: 14 4R/3Rs and 3 3R/3Rs). These half sibs [24.2 ± 3.5 months of age (mean ± SD)] were shipped to one of five livestock markets from their farms of origin in Iwate Prefecture. The calves were born in the 2002, 2003, and 2004 February-to-May calving seasons (spring) in their respective farmers’ feedlots. Bull calves were castrated at approximately 4–5 months after birth. The calves were grazed in regional public ranches with their dams from May to October each year before being returned to their respective farmers’ feedlots for finishing. The calves had restricted access to concentrate feeds and *ad libitum* roughage intake until shipping in 2004, 2005, and 2006. The roughage type in public ranches and farmers’ feedlots varied between rearing places. The general feeding conditions for fattening were as follows: (1) preceding term (8–16 months of age), 1.4% of BW concentrate intake per day and grass silage *ad libitum*; (2) midterm (17–20 months of age), 1.8% of BW concentrate intake per day and 2 kg/d of paddy straw; and (3) later term (more than 20 months of age), 8–10 kg/d of concentrate and 2 kg/d of paddy straw. After slaughter, the carcasses were dissected and evaluated at the sixth and seventh rib sections according to the Japanese meat grading system by a certified grader of the Japan Meat Grading Association (1988). The traits measured included carcass weight (CW; kg), *longissimus* muscle area (LMA; cm^2^), rib thickness (RT; cm), subcutaneous fat thickness (SFT; cm), and firmness, texture, and beef marbling score number (BMS No.). Marbling was scored from 1 (poor) to 12 (very abundant) according to the beef marbling standard (Japan Meat Grading Association 1988).

### Statistical analysis

A mixed-inherited animal model was used to evaluate the effect of genotype and to compute basic summary statistics on traits of interest. The snp_ad, snp_a, snp_d, and epi_snp options of the Qxpak software (Pérez-Enciso and Misztal 2004) were used for analyzing the additive and dominant effects of the *DelR242* [or *nt-7(C > A)*] locus and for the effect of epistasis between the *DelR242* and *nt-7(C > A)* loci. The following models were used:

$$\text{Exp. 1}\;y_{ijkno}=year_{i}+area_{j}+bx_{k}+SNP+u_{o}+e_{ijkno}$$

$$\text{Exp. 2}\;y_{ijklmno}=year_{i}+area_{j}+bx_{k}+\mathrm{se}x_{l}+\mathrm{shippin}g_{m}+SNP+u_{o}+e_{ijklmno},$$

where *y_ijkno_* or *y_ijklmno_* is an individual phenotypic observation; the effect of year *i* (*i*, 14 or 3 levels for Exp. 1 or Exp. 2), the grazing area effect (*area_j_*) (*j*, 5 or 4 levels for Exp. 1 or Exp. 2), the sex effect (sex*_l_*) (*l*, 2 levels for Exp. 2), and the shipping month effect (shipping*_m_*) (*m*, 12 levels for Exp. 2) were used as fixed effects. *b* is a regression coefficient of age (*x_k_*) at the start of direct testing (Exp. 1) or of month of shipping (Exp. 2). In this analysis, the infinitesimal genetic effect was included and treated as a random effect (*u_o_*) with covariance matrix, Aσ_u_^2^ (where A is the numerator relationship matrix). *e_ijkno_* and *e_ijklmno_* are vectors of random residual effects. *SNP* is the single-locus SNP (including the *DelR242*) genotypic effect, which was partitioned into additive (*a*) and dominance (*d*) effects.

The likelihood ratio test was performed by removing the single-locus SNP genotypic effect from the model, and normal *P* values were obtained by assuming a χ^2^ distribution of the likelihood ratio test (degrees of freedom = 2).

The proportion of genotypic variance explained by the model was calculated as follows:

$$\mathrm{Variance}\;\mathrm{ratio}=\left[2pq{\left(a+d\left(q-p\right)\right)}^{2}\right]/V_{A}$$

The ratio of the variance of dominance deviation to the additive genetic variance of the trait was calculated as follows:

$$\mathrm{Variance}\;\mathrm{ratio}=\left[{\left(2pqd\right)}^{2}\right]/V_{A},$$

where *p* and *q* are allelic frequencies, *a* and *d* are additive and dominant effects of the *DelR242* locus, respectively, and *V_A_* is the additive genetic variance of the trait obtained from an animal model ignoring single-locus SNP genotypic effects (Falconer and Mackay 1996).

For least-squares means analysis, we performed analyses of variance (ANOVAs) with the generalized linear model (GLM) procedure in SAS (SAS 2004) fitting the fixed effects of genotype (Exp. 1 and Exp. 2), test year (Exp. 1 or Exp. 2), grazing area (Exp. 1 or Exp. 2), sex (Exp. 2), shipping month (Exp. 2), sire (17 levels for Exp. 2), and a linear covariate of age of animals on testing (Exp. 1) or shipping (Exp. 2). The significance threshold was set to a Bonferroni-corrected *P* depending on the number of traits in Exp. 1.

### Linkage disequilibrium (LD) analysis

In total, 30 nucleotide variations (26 SNPs, 3 indels including *DelR242* and intron 1 microsatellite [*(GTTT)*_*5*_ or *(GTTT)*_*6*_] of 23 individuals (nine *Bos taurus* breeds; accession no.: AB492155–AB492177) were used to estimate local LD and haplotype blocks for a 5.8-kb genomic region that surrounds the *DelR242* locus of *GHSR1a*. The genotype data [*nt-7(C > A)*, *nt456(G > A)*, and *DelR242*] of 95 sires and 540 half sibs were also used to estimate local LD. Local LD (*r^2^*) and common haplotype patterns were computed with Haploview version 4.2 (Barrett *et al.* 2005).

## Results

### Differences in allele and haplotype frequencies between sires and dams

The frequencies of the *3R* allele of the *DelR242* locus in the 17 sires of 540 half sibs and in the 540 half sibs were significantly higher than those in the 95 sire and 540 dam haplotypes. Differences in the allele frequencies of the *DelR242*, *nt-7(C > A)*, *nt456(G > A)*, and *5′UTR-(TG)_n_* loci between 17 sires and 540 half sibs were not significant (Table S2). Accordingly, allele and haplotype frequencies of the *DelR242*, *nt-7(C > A)*, *nt456(G > A)*, and *5′UTR-(TG)_n_* loci were compared between 95 sires (as “sires”) and 540 dam haplotype (as “dams”) (Table 1). It was evident that the frequencies of the *3R* (*DelR242)*, *C* [*nt-7(C > A)*], and *A* [*nt456(G > A)*] alleles in sires were significantly higher than those in dams (*P* < 0.03, *P* < 0.05, and *P* < 0.05, respectively). The χ^2^ values for the *nt456(G > A)* and *nt-7(C > A)* loci were lower than that for the *DelR242* locus. There was no significant difference between sires and dams in allele frequencies of the *5′UTR-(TG)_n_* locus (*P* < 0.10). In comparison, it was evident that the haplotype frequencies of the *C-3R* ([*nt-7(C > A)*]-[*DelR242*] haplotype) and the *A-3R* ([*nt456(G > A)*]-[*DelR242*] haplotype) in sires were significantly higher than those in dams (*P* < 0.05 and *P* < 0.02, respectively). There was no significant difference between sires and dams in the [*nt-7(C > A)*]-[*nt456(G > A)*] and [*5′UTR-(TG)_n_*]-[*nt-7(C > A)*]-[*DelR242*] haplotype frequencies (*P* < 0.10 and *P* < 0.10, respectively).

**Table 1:** Allele frequencies and haplotype frequencies in sires and dams in Japanese Shorthorn cattle

Locus Allele or Haplotype	Sires	Dams	Frequency Difference Between Sires and Dams		
	(190)*^a^*	(540)*^b^*	χ^2^	df*^c^*	*P*
*DelR242*			5.2	1	<0.03
*4R*	0.595	0.686			
*3R*	0.405	0.314			
*nt-7(C > A)*			4.6	1	<0.05
*A*	0.732	0.806			
*C*	0.268	0.194			
*nt456 (G > A)*			4.3	1	<0.05
*A*	0.410	0.327			
*G*	0.590	0.673			
*5′UTR-(TG)_n_*			15.2	9	<0.10
*19-TG**^d^*	0.016	0.017	0.0	1	
*20-TG*	0.195	0.264	3.6	1	
*21-TG*	0.011	0.013	0.1	1	
*22-TG*	0.116	0.201	6.9	1	<0.01
*23-TG*	0.211	0.156	3.0	1	
*24-TG*	0.068	0.045	1.5	1	
*25-TG*	0.153	0.135	0.4	1	
*26-TG*	0.037	0.026	0.6	1	
*28-TG*	0.179	0.135	2.2	1	
*31-TG*	0.016	0.007	1.2	1	
[*nt-7(C > A)*]-[*DelR242*]			8.2	3	<0.05
*A-4R*	0.495	0.608	7.4	1	<0.01
*C-4R*	0.095	0.078	0.5	1	
*A-3R*	0.237	0.198	1.3	1	
*C-3R*	0.174	0.116	4.2	1	<0.05
[*nt456(G > A)*]-[*DelR242*]			7.9	2	<0.02
*A-4R*	0.000	0.013	2.5	1	
*G-4R*	0.589	0.673	4.4	1	<0.05
*A-3R*	0.411	0.314	5.9	1	<0.05
[*nt-7(C > A)*]-[*nt456(G > A)*]			7.0	3	<0.10
*A-A*	0.237	0.183	2.6	1	
*C-A*	0.174	0.152	0.5	1	
*A-G*	0.494	0.600	6.5	1	<0.05
*C-G*	0.095	0.065	1.9	1	
[*5′UTR-(TG)_n_*]-[*nt-7(C > A)*]-[*DelR242*]			30.7	21	<0.10
[*19-TG*]*-A-4R*	0.016	0.013	0.1	1	
[*19-TG*]*-A-3R*		0.004	0.7	1	
[*20-TG*]*-A-4R*	0.189	0.258	3.6	1	
[*20-TG*]*-A-3R*		0.004	0.7	1	
[*20-TG*]*-C-4R*	0.005	0.002	0.6	1	
[*21-TG*]*-A-4R*	0.011	0.013	0.1	1	
[*22-TG*]*-A-4R*	0.089	0.180	8.7	1	<0.005
[*22-TG*]*-A-3R*		0.004	0.7	1	
[*22-TG*]*-C-4R*	0.026	0.018	0.5	1	
[*23-TG*]*-A-3R*	0.032	0.031	0.0	1	
[*23-TG*]*-C-4R*	0.005	0.011	0.5	1	
[*23-TG*]*-C-3R*	0.174	0.114	4.5	1	<0.05
[*24-TG*]*-A-4R*	0.011		5.7	1	<0.05
[*24-TG*]*-C-4R*	0.058	0.045	0.5	1	
[*25-TG*]*-A-4R*	0.153	0.131	0.5	1	
[*25-TG*]*-A-3R*		0.002	0.4	1	
[*25-TG*]*-C-4R*		0.002	0.4	1	
[*26-TG*]*-A-4R*	0.026	0.013	1.6	1	
[*26-TG*]*-A-3R*	0.011	0.013	0.1	1	
[*28-TG*]*-A-3R*	0.179	0.135	2.2	1	
[*31-TG*]*-A-3R*	0.016	0.006	1.8	1	
[*31-TG*]*-C-3R*		0.002	0.4	1	

Allele frequencies of the *GHSR1a-DelR242, nt-7(C > A), nt456(G > A)*, and 5′UTR microsatellite [*5′UTR-(TG)_n_*] loci and haplotype frequencies of [*nt-7(C > A)*]-[*DelR242*], [*nt456(G > A)*]-[*DelR242*], [*nt-7(C > A)*]-[*nt456(G > A)*], and [*5UTR-(TG)_n_*]-[*nt-7(C > A)*]-[*DelR242*] in sires and dams in Japanese Shorthorn cattle.

a A total of 190 haplotypes derived from 95 sires.

b A total of 540 haplotypes derived from 540 dams of 540 half sibs. A dam’s allele or haplotype was estimated by removing the sire’s transmitted allele or haplotype from the half sib’s genotypes or haplotype combinations. The sire genotypes were 14 *4R/3R* heterozygotes and 3 *3R/3R* homozygotes.

c Degrees of freedom.

d (TG)_19_

### LD analysis in *GHSR1a*

**Figure 1:**
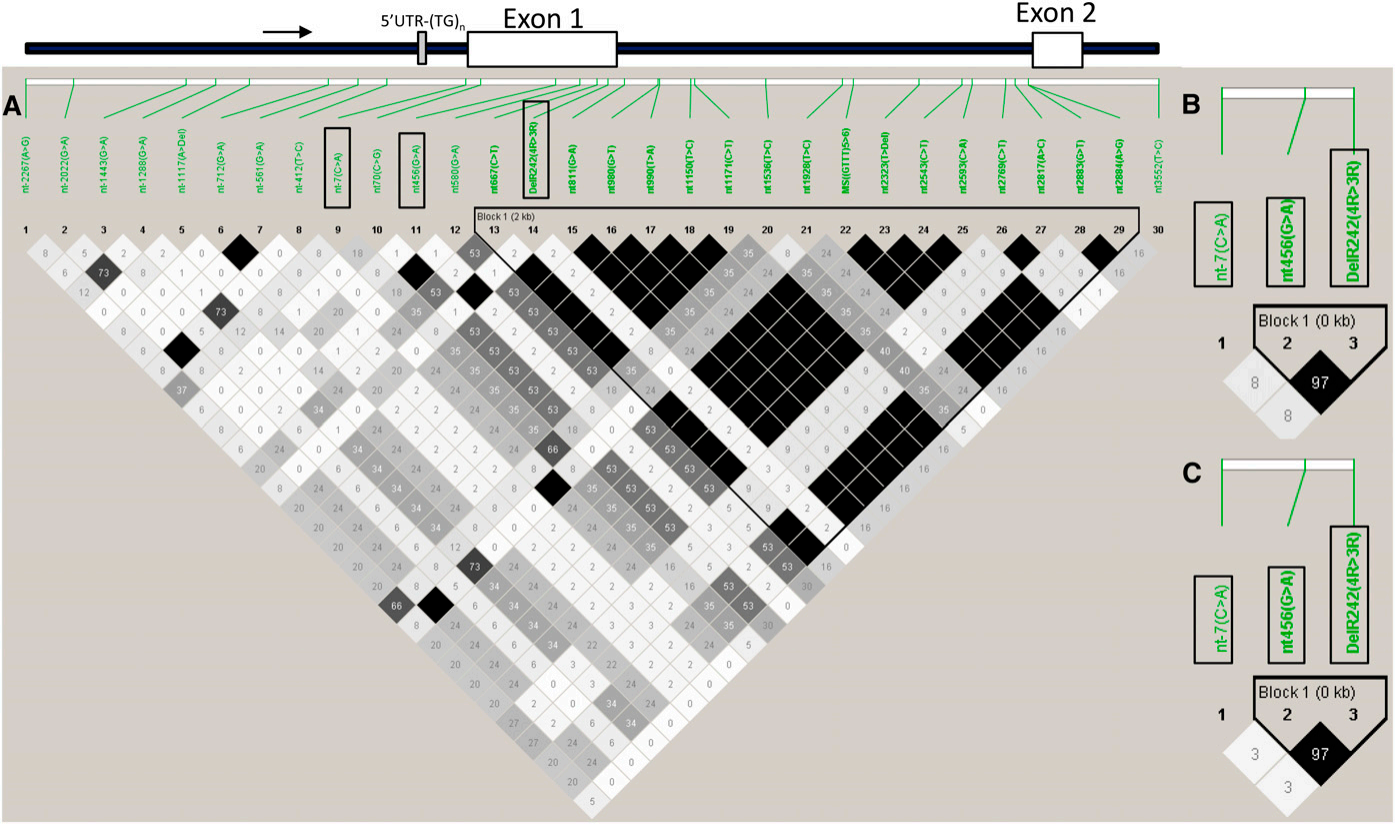
Summary of local LD and haplotype blocks in a 5.8-kb genomic region that surrounds the *DelR242* locus of *GHSR1a*. (A) Thirty nucleotide variations (26 SNPs, 3 indels including *Del242* and the intron 1 microsatellite) [*(GTTT)_5_* or *(GTTT)_6_*: *(GTTT)_5 > 6_*] of 23 individuals (accession no. AB492155–AB492177). (B and C) Three nucleotide variations (*nt-7(C > A)*, *nt456(G > A)*, and *DelR242*) of 95 sires and 540 half sibs. LD plot generated in Haploview (Barrett *et al.* 2005) indicates *r^2^* values between pairs of SNPs multiplied by 100; white, *r^2^* = 0; shades of gray, 0 < *r^2^* < 1; black, *r^2^* = 1. The *nt-7(C > A)*, *nt456(G > A)*, and *DelR242* loci are indicated with black squares. Transcription start regions are shown with black arrows. *5′UTR-(TG)_n_*, 5′UTR microsatellite.

We analyzed local LD and common haplotype patterns in a 5.8-kb genomic region around the *DelR242* locus in *GHSR1a*. We found a haplotype block of approximately 2 kb from the *nt667(C > T)* in exon 1 to the *nt2884 (A > G)* in intron 1 (Figure 1A). In addition, local LD analysis clearly showed that the *DelR242* locus is not in LD with *nt-7(C > A)*, the *5′UTR-(TG)_n_*, and the other 27 loci within *GHSR1a*, except for the *nt456(G > A)* locus. *DelR242* is in LD with the *nt456(G > A)* locus (*r^2^* = 1) (Figure 1 A). The strong LD between *DelR242* and *nt456(G > A)* was confirmed in 95 sires and 540 half sibs (Figures 1, B and C). It was observed that *nt70(C > G)*, *nt580(G > A)*, *nt667(C > T)*, and intron 1 microsatellite were fixed for the homozygous genotypes [*C/C*, *G/G*, *C/C*, and *(GTTT)_5_/(GTTT)_5_*, respectively] in the 540 half sibs. The 15 markers in intron 1 were considered as fixed for homozygotes on the basis of the result of local LD analysis in Japanese Shorthorn cattle (Figure 1). *nt456(G > A)* is a synonymous nucleotide substitution in exon 1.

### Dominance/overdominance or additive effects of the *3R* allele of the *DelR242* locus

Table 2 and Table 3 show the summary of statistics, least-squares means of the *DelR242* genotypes (Table 2), and dominance/overdominance or additive effects of the *3R* allele of the *DelR242* locus on growth, feed efficiency, and BSCM traits in direct-tested bulls, as well as on carcass traits from shipped half sibs using mixed-inherited animal model analysis (Table 3). In direct-tested bulls, the *3R* allele had a significant dominance effect on ADG in both least-squares and mixed-inherited animal model analyses. The dominance effect was caused by overdominance. The effects of the *DelR242* locus showed no significant association with the seven carcass traits from shipped half sibs. The results indicate a significant overdominance effect in the direct-tested bulls. The *4R/3R* heterozygous individuals showed significantly higher ADG than the *4R/4R* and *3R/3R* homozygous individuals. The *4R/3R* heterozygous individuals showed the highest relative growth rates of BW (BW_RGR) and CW (CW_RGR) during the direct-testing period among three genotype individuals (Table S3).

**Table 2:** Least-squares means of the effects of the *DelR242* genotypes and conformation measurement traits

Traits	Units	Abbreviation	No. of Animals	Mean	SD*^b^*	Least-Squares Mean						*P* and Significance Level*^d^*	
						*4R/4R*	(SD)	*4R/3R*	(SD)	*3R/3R*	(SD)		
(1) Direct-tested bulls						(33)*^c^*		(61)		(27)			
Age at the start of direct testing	d	AGE	121	245.1	20.6	251.70	(18.5)	242.30	(22.5)	243.60	(17.2)	—	
Average dairy gain	kg/d	ADG	121	1.28	0.14	1.24*^c^*	(0.14)	1.33*^a^*	(0.1)	1.21*^c^*	(0.12)	0.000	***
180-d adjusted body weight	kg	180BW	121	242.2	30.8	238.2	(34.5)	242.4	(30.8)	247	(26.3)	0.473	
365-d adjusted body weight	kg	365BW	121	480.6	37.7	469.7*^b^*	(40.2)	490.2*^a^*	(36.4)	472.1*^a^*,*^b^*	(32.9)	0.005	^†^
Total thickness of 8 points of subcutaneous fat	mm	8SFT	121	79.9	15.7	79.0	(16.5)	82	(15.1)	76.3	(15.9)	0.049	
Roughage intake	%	ROUGH	121	45.0	2.3	44.4	(2.1)	45.0	(2.4)	45.6	(2.0)	0.072	
Roughage weights per kg body weight gain	kg	ROUGH_1	121	3.80	0.49	3.77*^c^*	(0.51)	3.68*^c^*	(0.39)	4.12*^a^*	(0.55)	0.000	**
Concentration weights per kg body weight gain	kg	CONC_1	121	4.56	0.54	4.70*^a^*	(0.57)	4.39*^c^*	(0.46)	4.79*^a^*	(0.58)	0.000	**
Body shape and conformation measurements													
Withers height at the start of direct testing	cm	WH_S	121	115.2	3.5	115.1	(2.9)	115.3	(3.8)	115.0	(3.7)	0.905	
Withers height at the end of direct testing	cm	WH_E	121	127.1	3.3	126.7	(3.1)	127.2	(3.4)	127.5	(3.4)	0.554	
						(25)*^c^*		(47)		(22)			
Chest width at the start of direct testing	cm	CW_S	94	38.6	3.7	40.2	(3.9)	37.9	(3.6)	38.6	(3.4)	0.023	
Chest width at the end of direct testing	cm	CW_E	94	46.8	2.9	46.4	(2.9)	47.2	(3.0)	46.4	(3.0)	0.504	
Rump length at the start of direct testing	cm	RL_S	94	42.5	2.3	42.9	(1.8)	42.7	(2.4)	41.7	(2.3)	0.053	
Rump length at the end of direct testing	cm	RL_E	94	48.9	2.2	48.8	(1.9)	49.1	(2.3)	48.7	(2.4)	0.720	
Cannon circumference at the start of direct testing	cm	CC_S	94	17.7	0.8	17.6	(0.9)	17.8	(0.6)	17.4	(0.8)	0.079	
Cannon circumference at the end of direct testing	cm	CC_E	94	19.8	0.7	19.9	(0.8)	19.9	(0.7)	19.7	(0.8)	0.605	
(2) Shipped half sibs*^a^*						(148)*^c^*		(283)		(106)			
Slaughter age	mo	—	537	24.2	3.5	24.2	(3.5)	24.2	(3.6)	24.0	(3.0)	—	
Carcass weight	kg	CW	537	407.0	38.0	406.3	(39.8)	406.5	(38.4)	409.0	(34.6)	0.567	
Longissimus muscle area	cm^2^	LMA	537	48.1	5.3	48.3	(5.6)	47.9	(5.0)	48.3	(5.4)	0.696	
Rib thickness	cm	RT	537	6.38	0.65	6.36	(0.68)	6.36	(0.62)	6.47	(0.67)	0.338	
Subcutaneous fat thickness	cm	SFT	537	2.39	0.66	2.41	(0.64)	2.38	(0.66)	2.38	(0.69)	0.800	
Beef marbling score		BMS	537	2.10	0.28	2.08	(0.25)	2.12	(0.35)	2.12	(0.27)	0.914	
Firmness			537	2.09	0.29	2.09	(0.29)	2.08	(0.29)	2.08	(0.28)	0.596	
Texture			537	2.38	0.49	2.35	(0.48)	2.34	(0.48)	2.44	(0.50)	0.286	

Least-squares means of the effects of the *DelR242* genotypes on growth, feed intake, and body shape and conformation measurement traits in direct-tested bulls and carcass traits in shipped half sibs.

a 209 dams and 328 steers.

b SD.

c Number of animals.

d *P* and significance level (Bonferroni correction): *P = 0.05/15 = 0.0033; **P = 0.01/15 = 0.00067; ***P = 0.001/15 = 0.000067.15; ^†^*P* = 0.10/15 = 0.0067. Number of traits for direct-tested bulls = 15. *P < 0.05; **P < 0.01, ***P < 0.001; ^†^*P* < 0.10, *^a,b^P* < 0.05; *^a,c^P* < 0.01. The direct testing duration is 140 d.

**Table 3:** Additive and dominance effects of the *3R* allele of the *DelR242* locus on growth, feed intake, and body shape and conformation measurements traits in direct-tested bulls and carcass traits in shipped half sibs

Traits (Abbreviation)*^a^*	Units	No. of Animals*^b^*	*V_A_^c^*	*h^2^^d^*	Effect of the *3R* Allele									
					LRT*^e^*	*P* and Significance Level*^f^*		Additive Effect [a]*^g^*	(SE)	Dominant Effect [d]*^g^*	(SE)	Ratio of *Va^h^* (%)	Ratio of *Vd^i^* (%)	Degree of Dominance [|d/a|]
(1) Direct-tested bulls														
ADG	kg/d	121	0.01	0.37	12.55	0.002	*	0.01^ns^	(0.02)	0.08**	(0.02)	1.6	30.0	8.9
180BW	kg	121	289.9	0.47	0.21	0.901		−1.15		1.99		—	—	1.7
365BW	kg	121	543.05	0.51	7.03	0.030		−0.72		16.40		0	12.3	22.8
8SFT	mm	121	50.04	0.52	1.26	0.533		−1.62		0.58		—	—	0.4
ROUGH	%	121	1.29	0.44	10.27	0.006	^†^	0.77**	(0.25)	−0.48^ns^	(0.32)	21.3	5.7	0.6
ROUGH_1	kg	121	0.06	0.31	10.45	0.005	^†^	0.10^ns^	(0.06)	−0.25**	(0.08)	5.9	26.4	2.6
CONC_1	kg	121	0.07	0.36	6.51	0.039		−0.05		−0.21		—	—	—
Body shape and conformation measurements														
WH_S	cm	121	4.33	0.59	3.57	0.165		−0.37		0.95		—	—	—
WH_E	cm	121	4.18	0.56	1.74	0.419		−0.04		0.70		—	—	—
CW_S	cm	94	3.8	0.51	4.36	0.113		−0.77		−0.78		—	—	—
CW_E	cm	94	3.22	0.49	0.08	0.960		0.08		−0.13		—	—	—
RL_S	cm	94	1.97	0.66	6.07	0.048		−0.64		0.30		—	—	—
RL_E	cm	94	1.00	0.34	0.08	0.960		−0.10		0.16		—	—	—
CC_S	cm	94	0.23	0.39	9.67	0.008		−0.20		0.47		—	—	—
CC_E	cm	94	0.21	0.49	5.28	0.071		−0.20		0.21		—	—	—
(2) Shipped half sibs														
CW	kg	537	542.85	0.50	1.16	0.561		1.32		−2.54		—	—	—
LMA	cm^2^	537	10.91	0.44	0.72	0.696		0.22		−0.23		—	—	—
RT	cm	537	0.16	0.40	2.17	0.338		0.05		−0.05		—	—	—
SFT	cm	537	0.17	0.45	0.45	0.800		−0.02		−0.03		—	—	—
BMS		537	0.05	0.42	0.18	0.914		0.01		0.01		—	—	—
Firmness		537	0.03	0.40	1.03	0.596		−0.02		−0.01		—	—	—
Texture		537	0.09	0.39	2.5	0.286		0.03		−0.06		—	—	—

a For full names of the traits, see Table 2.

b Total number of the 3 *DelR242* genotype individuals (see Table 2).

c Additive genetic variance.

d Heritability.

e Likelihood ratio test.

f *P* and significance level (with Bonferroni correction): *P = 0.05/15 = 0.0033; ^†^*P* = 0.10/15 = 0.0067; 15 = number of the traits for direct tested bulls. *P < 0.05; ^†^*P* < 0.10.

g Additive effect [a] and dominance effect [d] of the *3R* allele. These effects were estimated by the snp_ad option. The significance levels for the additive effect [a] were estimated by the snp_a option and those for the dominance effect [d] were measured by the snp_d option: **P < 0.01; ns, not significant.

h Proportion of the additive variance of the *3R* allele to additive variance of the trait (*V_A_*).

i Ratio of the variance of dominance deviation of the *3R* allele to *V_A_*.

## Discussion

### Sex difference and high frequency of the *3R* allele in Japanese Shorthorn cattle

Table 2 and Table 3 clearly show that a *4R/3R* heterozygote weaner bull has a selective advantage over its homozygous peer in direct testing because of a heterozygous advantage of the *DelR242* locus effect on ADG. The selection index formula for direct testing of Japanese Shorthorn bull is as follows (*Materials and Methods* in File S2):

[21.749×ADG of direct testing period]−[0.254×8SFT]+10

The aggregate breeding value formula for progeny testing of direct-tested bulls is as follows:

$$\begin{array}{l} \left[11.1\mathrm{\times breeding}\;\mathrm{value}\;\mathrm{of}\;\mathrm{ADG}\;\mathrm{at}\;\mathrm{direct}\;\mathrm{testing}\right]-[0.031\mathrm{\times breeding}\;\mathrm{value}\;\mathrm{of}\;8\mathrm{SFT}\;\mathrm{at}\;\mathrm{direct}\;\mathrm{testing}]+[4.67\times \mathrm{breeding}\;\mathrm{value}\;\mathrm{of}\;\mathrm{ADG}\;\mathrm{at}\;\mathrm{progeny}\;\mathrm{testing}]+\left[0.025\mathrm{\times REA}\;\mathrm{at}\;\mathrm{progeny}\;\mathrm{testing}\right]+[1.69\mathrm{\times breeding}\;\mathrm{value}\;\mathrm{of}\;\mathrm{SFT}\;\mathrm{at}\;\mathrm{progeny}\;\mathrm{testing}]+\left[0.807\mathrm{\times BMS}\;\mathrm{at}\;\mathrm{progeny}\;\mathrm{testing}\right] \end{array}$$

These formulas show that ADG is the most important trait in the selection of Japanese Shorthorn sires. At present, the *DelR242* genotypes of 11 core sires for insemination at IARC are seven *4R/3R* heterozygotes and four *3R/3R* homozygotes. The expected ratios of *4R/3R* heterozygous individuals in progeny produced by mating of the progeny-tested sire with dams in 0.3 of the *3R* allele frequency in the dam population are 30% for *4R/4R*, 50% for *4R/3R*, and 70% for *3R/3R* sires (Table S4). These ratios could explain the significant sex differences between sires and dams and the higher *3R* allele frequency in Japanese Shorthorn cattle than in other cattle breeds. The *3R* allele of the *DelR242* locus has been reported to be widely distributed at low frequency in both the *B. taurus* and *B. indicus* cattle breeds (Komatsu *et al.* 2010), strengthening earlier assumptions that the original herd of Japanese Shorthorn cattle had a low frequency of the *3R* allele. Consolidation of the selective advantage of the *4R/3R* heterozygous weaner bulls would have the potential to increase the frequency of the *3R* allele in the sire population and, subsequently, in the dam population of Japanese Shorthorn cattle. Because of the relatively small number of direct-tested bulls in this study, further investigations of the overdominance effect of the *DelR242* locus on growth in weaner bulls in other herds and/or other beef cattle breeding populations carrying the *3R* allele are required.

### Overdominance effect of the *DelR242* single locus

Exploitation of heterosis has become a major strategy for increasing the productivity of animals and plants. Dominance-based (Bruce 1910), overdominance-based (East 1936), and pseudo-overdominance/epistasis-based models (Stuber *et al.* 1973; Stuber *et al.* 1992) are commonly used to explain heterosis. The dominance model is based on the hybridization of two inbred parental lines to exploit breed complementarity in the F_1_ progeny by superior alleles at different loci, resulting in a phenotypic improvement over the mean of the parents. The overdominance model is based on interactions between the alleles originating from each parent at each locus that give rise to intralocus synergistic (nonlinear) effects on growth or other traits. The pseudo-overdominance model operates when two or more dominant or recessive mutations in different parents are linked or “in repulsion.” The epistasis model is based on nonallelic interactions between two or more loci that lead to superior phenotypic expression in hybrids (Goff and Zhang 2013). Gemmell and Slate (2006) provided five new examples of heterozygote advantage, or overdominance, on the basis of polymorphism in the oocyte-derived growth factor and growth differentiation factor of nine genes that affect female fecundity in domesticated sheep.

It is important to determine whether the overdominance effect is controlled by the *DelR242* single locus or other linked loci or by epistasis with other loci within *GHSR1a*.

Genome-wide association studies (GWAS) have identified areas of the genome associated with feed intake and growth traits in beef cattle (Rolf *et al.* 2011; Lu *et al.* 2013; Saatchi *et al.* 2014). One of the 66 SNPs markedly associated with residual feed intake is located at 85.95 Mb (Rolf *et al.* 2011) on BTA1 and 1 of the 36 SNPs associated with dry matter intake (DMI) is located at 89.36 Mb on BTA1 (Lu *et al.* 2013). One of the six large-effect quantitative trait loci (QTL) associated with feedlot DMI is located at 107 Mb and 1 of the 11 large-effect QTL associated with mid-test BW is located at 98 Mb on BTA1 in the Angus cattle breed (Saatchi *et al.* 2014). *GHSR1a* is located at 97.14 Mb on BTA1. Lindholm-Perry *et al.* (2011) reported an association between SNP located at 97.25 Mb and average daily feed intake (ADFI) and between SNP located at 98.72 Mb and ADG on BTA1 in a crossbred beef cattle population used in the original GWAS (Snelling *et al.* 2010). They did not find an association between the *3R* allele substitution effect and ADFI or ADG. However, these authors did not estimate the dominance effect of the *DelR242* locus on these traits. These studies suggest that one QTL associated with an overdominance effect on growth or feed intake in cattle is located within or adjacent to the *GHSR1a* region.

MicroRNAs (miRNAs) are small (approximately 22 nucleotides long) endogenous noncoding molecules that can play important regulatory roles in animals and plants by targeting mRNAs for cleavage or translational repression. miRNAs mainly target complementary sites of 3′UTRs of mRNAs (Bartel 2004). We previously compared the 3′UTR sequences of *GHSR1a* among 10 haplotypes (Hap01–Hap10: Hap01–Hap08, the *4R* type; Hap09 and Hap10, the *3R* type; accessions AB492155–AB492175) and found no differences between haplotypes (Komatsu *et al.* 2010). This result suggests that there are no differences between the *4R* and *3R* alleles in the allele-specific expression (ASE) of the *GHSR1a* transcripts that are attributable to miRNA regulation.

The *19-TG* allele of the *5′UTR-(TG)_n_* locus and the *A* allele of the *nt-7(C > A)* locus have been reported to show significant desirable additive effects on CW and ADG in Japanese Black cattle (Komatsu *et al.* 2011). Epistatic effects between the *DelR242* and *nt-7(C > A)* loci on the traits in direct-tested bulls and shipped half sibs were not significant (Table S5). Furthermore, the *nt-7(C > A)* locus showed neither additive nor overdominance effects on these traits in direct-tested bulls (Table S5).

Taken together, these results suggest that the overdominance effect of the *DelR242* locus on ADG observed in weaner bulls may be controlled by the *DelR242* single locus. Further investigations of GWAS and epistatic effects between *DelR242* and other loci are required to shed light on the single overdominance effect of the *DelR242* locus on ADG in other Japanese Shorthorn herds.

### Age-dependent overdominance effect of the *DelR242* locus

Significant overdominance effects of the *DelR242* locus on ADG were confined to the direct testing period (approximately 8 months of age) (Table 2 and Table 3). Percentage heterosis of BW and daily gains from birth to 13 months of age were the highest during the early postweaning period, similar to the period of 7–8 months of age in bulls from five inbred lines of Hereford cattle (Urick *et al.* 1968).

GH release in response to stimulation by ghrelin and GHRH is age-dependent, and episodic GH release is higher in postweaning than in preweaning calves and cows (Itoh *et al.* 2005). A positive correlation between the area under the GH response curve (AUC-GH) to GHRH and ADG was observed in Angus weaner bulls (average age: 229 d) (Connor *et al.* 1999). In cattle, *GHSR1a* mRNA expression was reported to be higher in the arcuate nucleus, pituitary gland, and liver than in other tissues, and *GHSR1a* mRNA expression in the arcuate nucleus of postweaning calves was 10-fold greater than that in preweaning calves and cows (Komatsu *et al.* 2012). Ghrelin stimulates GH secretion, primarily by binding to GHSR1a on GHRH neurons in the arcuate nucleus of the hypothalamus (Mano-Otagiri *et al.* 2006). Transgenic mice overexpressing human GHSR1a in GHRH neurons showed increased hypothalamic GHRH expression, pituitary GH contents, and postweaning growth rates. However, BWs of the transgenic mice became similar to those of the wild-type mice in adulthood (Lall *et al.* 2004). Although we did not evaluate plasma GH concentrations or the additive, dominance, and overdominance effects of the *DelR242* locus on the growth of bulls after direct testing through to adulthood, the results of the present study suggest that the overdominance effect of the *DelR242* locus on growth is directly controlled by the highest episodic GH release in postweaning calves, resulting from the highest expression of *GHSR1a* mRNA in the arcuate nucleus.

### A possible molecular mechanism and hypothesis for the *DelR242* single-locus overdominance effect in weaner bulls

**Figure 2:**
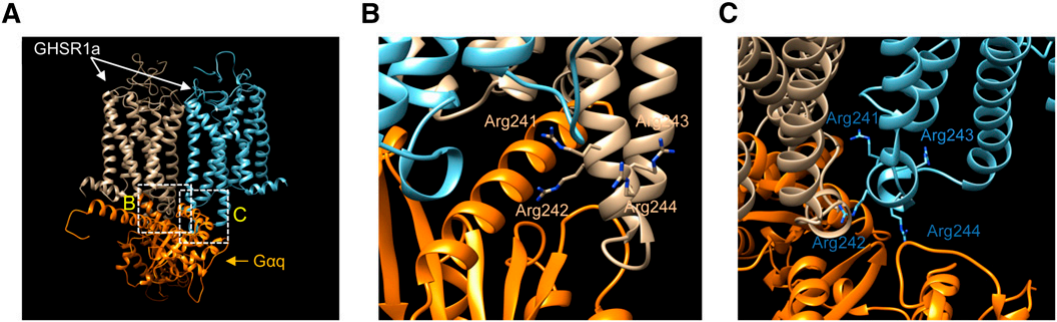
Ribbon representation of a model structure of the GHSR1a(4R) dimer–Gαq complex. The two GHSR1a(4R) protomers are brown and light blue, and Gαq is orange. (A) Entire view. (B) Close-up view of the interface between 1 GHSR1a(4R) protomer and Gαq. Side-chain nonhydrogen atoms of the arginine residues of the 4R region of the GHSR1a(4R) protomer (Arg241, Arg242, Arg243, and Arg244) are shown in a stick representation. Nitrogen atoms are blue. Carbon atoms are colored in the same color as that of the ribbon. (C) Close-up view of the interface between the other GHSR1a(4R) protomer and Gαq. Arginine residues of the 4R region of the GHSR1a(4R) protomer are shown as in (B). Images were generated with UCSF Chimera (Pettersen *et al.* 2004).

We considered a possible molecular mechanism of the overdominance effect caused by the *DelR242* locus. We built a model structure of the GHSR1a dimer–Gαq complex on the basis of the suggestion by Holst *et al.* (2005) that GHSR1a forms a homodimer (see *Materials and Methods* in File S1). We found that the 4R regions of the two GHSR1a protomers simultaneously interacted with Gαq at different sites when GHSR1a was dimerized via interactions between transmembrane helices 5 and 6 (TM-V and TM-VI) (Figure 2 and Figure S2). Deletion of an arginine residue from each 4R region will markedly change the structure of the region and the interactions with Gαq. Although it is difficult to predict how the Gαq-activation ability of the GHSR1a dimer is affected by deletion from the model structure, it is possible that the interactions between the GHSR1a(4R)–GHSR1a(3R) or GHSR1a(3R)–HSR1a(4R) “hetero”-dimer and Gαq are more favorable for the activation of Gαq than the interactions with GHSR1a “homo”-dimers. Thus, the activation of intracellular signaling transduction in the GHRH neuron of the *4R/3R* heterozygotes becomes greater than that of the homozygotes.

## Supplementary Material

Supporting Information
