# Supplementary material for: Overdominance Effect of the Bovine Ghrelin Receptor (GHSR1a)-DelR242 Locus on Growth in Japanese Shorthorn Weaner Bulls: Heterozygote Advantage in Bull Selection and Molecular Mechanisms
Source: G3 (Bethesda). 2014 Dec 23;5(2):271–9. doi: 10.1534/g3.114.016105 (PMC4321035; doi:10.1534/g3.114.016105)
Supplement: Supporting Information [file supp_5_2_271__index.html]

Overdominance Effect of the Bovine Ghrelin Receptor (GHSR1a)-DelR242 Locus on Growth in Japanese Shorthorn Weaner Bulls: Heterozygote Advantage in Bull Selection and Molecular Mechanisms — Supporting Information 

# Overdominance Effect of the Bovine Ghrelin Receptor (*GHSR1a*)-*DelR242* Locus on Growth in Japanese Shorthorn Weaner Bulls: Heterozygote Advantage in Bull Selection and Molecular Mechanisms

## Supporting Information for Komatsu *et al.*, 2015

**Files in this Data Supplement:**

- Supporting Information - Files S1-S2, Figures S1-S3, and Tables S1-S5 (PDF, 760 KB)
- File S1 - Materials and Methods: Modeling of the GHSR1a-Gαq complex structure. (PDF, 134 KB)
- File S2 - Materials and Methods: Direct-testing and progeny-testing programs in Japanese Shorthorn bulls. (PDF, 144 KB)
- Figure S1 - Differential amino acid sequences of the GHSR1a protein among six mammalian species. (PDF, 397 KB)
- Figure S2 - Structure of the bovine Gαq subunit and amino acid residues within 6 Å from the non-hydrogen atoms of the 4R region of the GHSR1a monomer. (PDF, 183 KB)
- Figure S3 - The position of 11 body shape and conformation measurements traits (Japanese Society of Animal Science, 2001 *Animal Husbandry Terminology Dictionary* (New Version). YOHKENDO Inc. Tokyo, Japan). (PDF, 250 KB)
- Table S1 - Primers used for DNA amplification, fragment analyses and sequencing. (PDF, 199 KB)
- Table S2 - Allele frequencies of the *GHSR1a-DelR242, nt-7(C>A), nt456 (G>A), 5'UTR microsatellite* [*5'UTR-(TG)n*] loci and haplotype frequencies of the [*nt-7(C>A)*]-[*DelR242*], [*nt456(G>A)*]-[*DelR242*], [*nt-7(C>A)*]-[*nt456(G>A)*] and [*5'UTR-(TG)n*]-[*nt-7(C>A)*]-[*DelR242*] in 95 sires, 17sires of 540 half sibs, 540 half sibs, and 540 dam haplotypes of 540 half sibs in Japanese Shorthorn cattle. (PDF, 230 KB)
- Table S3 - Comparison of relative growth rates (RGR) of body shape and conformation traits among the *DelR242* genotypes in direct-tested weaner bulls. (PDF, 242 KB)
- Table S4 - Changes in the ratio of the *4R/3R* heterozygous individual in his progenies produced by mating between the progeny-tested sire and dams depending on the *3R* allele frequency in the dam population. (PDF, 233 KB)
- Table S5 - Summary statistics of growth, feed intake, body shape and conformation measurements traits in direct-tested bulls, carcass traits in shipped half sibs, additive and dominance effects of the *C* allele of the *nt-7(C>A)* locus and epistatic effect between the *nt-7(C>A)* and *DelR242* loci. (PDF, 233 KB)
