# Supplementary material for: Overdominance Effect of the Bovine Ghrelin Receptor (GHSR1a)-DelR242 Locus on Growth in Japanese Shorthorn Weaner Bulls: Heterozygote Advantage in Bull Selection and Molecular Mechanisms
Source: G3 (Bethesda). 2014 Dec 23;5(2):271–9. doi: 10.1534/g3.114.016105 (PMC4321035; doi:10.1534/g3.114.016105)
Supplement: Supporting Information [file supp_g3.114.016105_016105SI.pdf]

G3·Genes | Genomes | Genetics

Overdominance effect of the bovine ghrelin receptor (GHSR1a)-DelR242 locus on growth in Japanese Shorthorn weaner bulls: heterozygote advantage in bull selection and molecular mechanisms

Masanori Komatsu,[*,2] Yoichi Sato,[§,1] Tatsuki Negami,[†] Tohru Terada,[†,‡] Osamu Sasaki,[*] Jumpei Yasuda,[§] Aisaku Arakawa,[††] Chikara Yoshida,[§] Hideaki Takahashi,[*] Aduli E.O. Malau-Aduli,[**] Keiichi Suzuki,[§§] and Kentaro Shimizu[†,‡]

*Animal Breeding Research Group, NARO Institute of Livestock and Grassland Science, National Agriculture and Food Research Organization (NARO), Tsukuba, Ibaraki 305-0901, Japan, §Animal Industry Research Institute, Iwate Agricultural Research Center (IARC), Takizawa, Iwate 020-0173, Japan, †Department of Biotechnology, Graduate School of Agriculture and Life Sciences, The University of Tokyo, Tokyo 113-8657, Japan, ‡Agricultural Bioinformatics Research Unit, Graduate School of Agriculture and Life Sciences, The University of Tokyo, Tokyo 113-8657, Japan, **Animal Science & Genetics, Tasmanian Institute of Agriculture, School of Land & Food, Faculty of Science, Engineering and Technology, University of Tasmania, Hobart, Tasmania TAS 7001, Australia, and Veterinary & Biomedical Sciences, College of Health, Medical & Veterinary Sciences, James Cook University, Townsville, Queensland QLD 4811, Australia, §§Graduate School of Agriculture, Tohoku University, Sendai, Miyagi 981-8555, Japan, ††Animal Research Group, National Institute of Agrobiological Sciences, Ikenodai 2, Tsukuba, Ibaraki 305-0901, Japan

[1]Present address: Department of Animal Science, Iwate Agricultural Junior College, Kanegasaki, 029-4501, Japan.

[2]Corresponding author: NARO Institute of Livestock and Grassland Science, National Agriculture and Food Research Organization (NARO), 2 Ikenodai, Tsukuba, Ibaraki, 305-0901, Japan. E-mail: mkomatsu@affrc.go.jp

DOI: 10.1534/g3.114.016105

File S1

Materials and Methods: Modeling of the GHSR1a-Gαq complex structure.

Although the structure of the complex between a GPCR dimer and a G protein has not yet been determined experimentally, a crystal structure of the complex between a GPCR monomer and a G protein is available. Therefore, we took a two-step approach to the modeling of the GHSR1a-Gαq complex structure, where the structures of the GHSR1a monomer and the GHSR1a monomer-Gαq complex were first modeled, and then the GHSR1a monomer model structure was docked to the GHSR1a monomer-Gαq complex structure. The structure of the 4R type of the bovine GHSR1a monomer and that of the complex between the 4R type of bovine GHSR1a and Gαq were predicted by comparative modeling. The structure of bovine inactive rhodopsin (PDB ID: 2I35) was used as the template for modeling the GHSR1a monomer, whereas the structures of active opsin (PDB ID: 3CAP) and the complex of bovine β2 adrenergic receptor¬ and Gs (PDB ID: 3SN6) were used as the templates for modeling the GHSR1a-Gαq complex. The sequence alignments between GHSR1a (GenBank Protein ID: DAA33245.1) and rhodopsin, opsin or β2 adrenergic receptor were obtained using the AlignMe server, which considers the transmembrane region for sequence alignment (Stamm *et al*. 2013). The sequence of Gαq (Refseq ID: NP_001103472.1) was aligned to that of Gs with the BLOSUM62 matrix (Henikoff and Henikoff 1992), and a gap opening penalty of 12 and gap extension penalty of 1 were incorporated using a sequence-alignment tool of UCSF Chimera (Meng *et al*. 2006). The structural models were generated using the program Modeller (Sali and Blundell 1993). Next, docking models between the GHSR1a monomer and GHSR1a-Gαq complex were generated by the ZDOCK 3.0.2 program (Pierce *et al*. 2011). The tilt angles of the GHSR1a monomer with respect to the normal membrane surface and the offset along the normal of the center-of-mass position of the GHSR1a monomer from that of the GHSR1a in the GHSR1a-Gαq complex were calculated for each representative structure using the FiPD program (Casciari *et al*. 2006). We selected the models with tilt angles and offsets of less than 0.5 radians and 5.0 Å, respectively. The interactions within the model structures were examined to identify the dimer interface between the GHSR1a protomers. The model having the best ZDOCK score was selected as the final model.

REFERENCE

Casciari, D., M. Seeber, and F. Fanelli, 2006 Quaternary structure predictions of transmembrane proteins starting from the monomer: a docking-based approach. BMC Bioinformatics 7: 340.

Henikoff, S., and J. G. Henikoff, 1992 Amino acid substitution matrices from protein blocks. Proc. Natl. Acad. Sci. USA. 89: 10915-10919.

Meng, E. C., E. F. Pettersen, G. S. Couch, C. C. Huang, and T. E. Ferrin, 2006 Tools for integrated sequence-structure analysis with UCSF Chimera. BMC Bioinformatics 7: 339.

Pierce, B.G., Y. Hourai, and Z. Weng, 2011 Accelerating protein docking in ZDOCK using an advanced 3D convolution library. PLoS One 6: e24657.

Sali, A., and T. L. Blundell, 1993 Comparative protein modelling by satisfaction of spatial restraints. J. Mol. Biol. 234: 779-815.

Stamm, M., R. Staritzbichler, K. Khafizov, and L. R. Forrest, 2013 Alignment of helical membrane protein sequences using AlignMe. PLoS One 8: e57731.

File S2

Materials and Methods: Direct-testing and progeny-testing programs in Japanese Shorthorn bulls.

(1) Direct-testing program**:** The calves were born between February and May in their respective farmers' feedlots, and grazed in regional public ranches with their dams from May to the end of September. The preliminary selection based on their body shape and conformation measurements (BSCM) was carried out at 4~5 months of age (Figure S3). 15 weaner bulls at 6–7 months of age were selected from a pool of 350 weaner bulls and transported to the direct-testing station at the Animal Industry Research Institute, Iwate Agricultural Research Center (IARC). They were fed with concentrate and had *ad libitum* access to roughage with an allowance of 42 days for adjustment and acclimatization, followed by the standard 140 days of direct-testing prior to slaughter. The average age at the start of direct-testing was 245.1 ± 20.6 days (mean ± SD). Routine management of the animals involved fortnightly recording of body weight (BW) and BSCM traits at the start and end of direct-testing (see MATERIALS AND METHODS: Animals and data collection). 5 weaner bulls were selected from a group of 15 weaner bulls based on their selection indices for direct-testing, pedigree and BSCM data. The selection index formula used for direct-testing of Japanese Shorthorn bulls is:

[21.749 × ADG of direct-testing period] – [0.254 × 8SFT] + 10.

(2) Progeny-testing program**:** 5 selected young bulls were mated with ordinary cows and their progeny (more than 6 half sib steer calves) were performance-tested. They were allowed to suckle their dams in addition to being fed concentrates and timothy-grass hay until weaning. After weaning, they were moved to the grower's barn and reared until the attainment of 7–8 months of age. They were fed with the conventional grower ration with an allowance of 20 days for adjustment and acclimatization followed by the standard 308 days (44 weeks) progeny-testing duration prior to slaughter. Routine management of the animals involved the recording of body weight, BSCM traits, concentrate and roughage intakes every 4 weeks. Steers were weighed at the beginning and end (WT) of the testing period so that average daily gain (ADG) could be computed. Carcass data collected included slaughter weight (WS), cold carcass weight (CW), ADG, rib eye area (REA), rib thickness (RT), carcass yield estimate (YE), subcutaneous fat thickness (SFT), inter-muscular fat thickness (IFT) and beef marbling score (BMS). One bull was selected from 5 bulls based on their aggregate breeding value, pedigree and BSCM data for progeny-testing. The aggregate breeding value formula used for progeny-testing of directly-tested bulls is:

[11.1 × breeding value of ADG at direct-testing] – [0.031 × breeding value of 8SFT at direct-testing] + [4.67 × breeding value of ADG at progeny-testing] + [0.025 × REA at progeny-testing] + [1.69 × breeding value of SFT at progeny-testing] + [0.807 × BMS at progeny-testing].

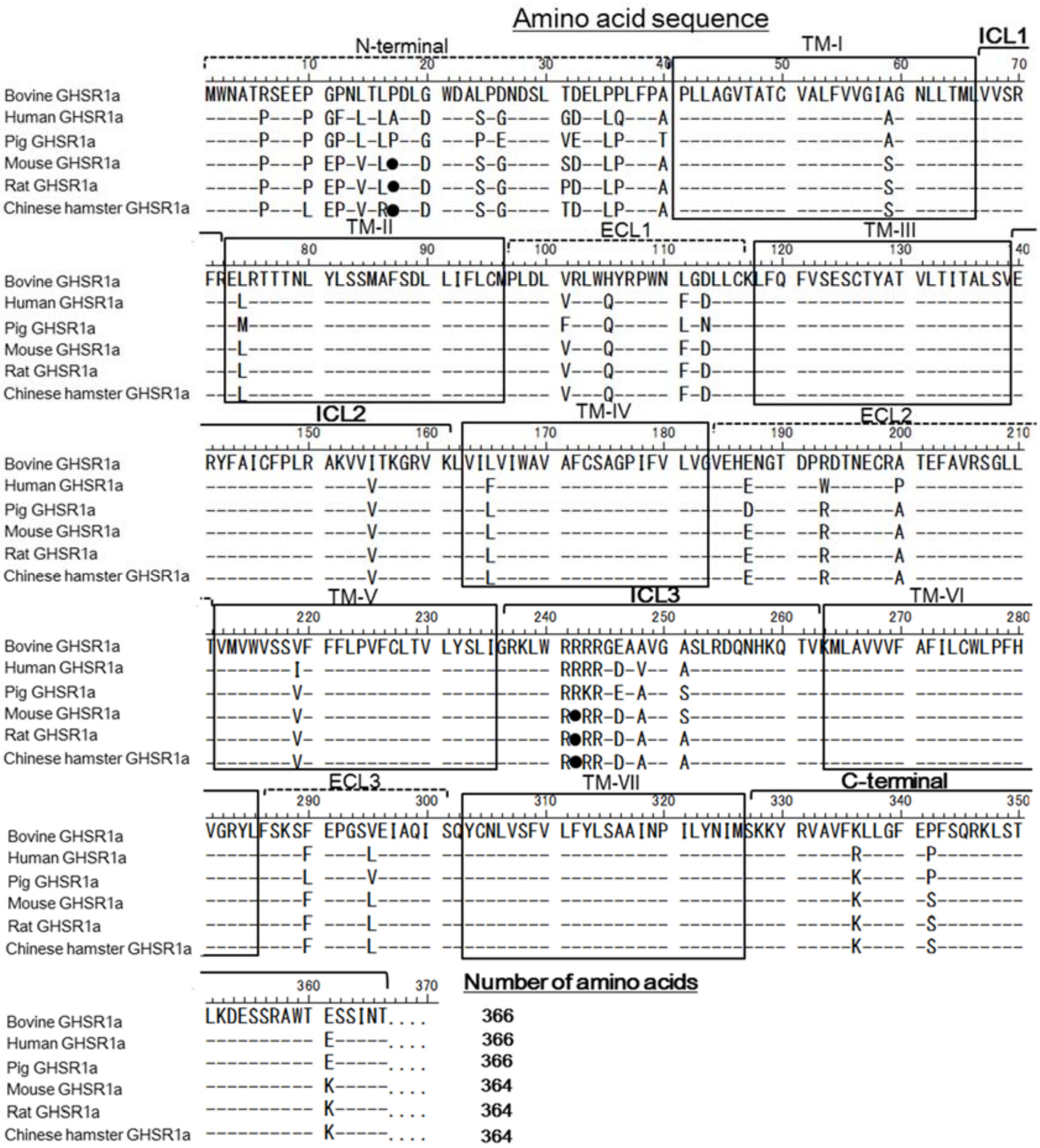

Figure S1 Differential amino acid sequences of the GHSR1a protein among six mammalian species. The dashes indicate identical residues. Black circles represent amino-acid deletions. The transmembrane helices are shown in boxes (TM-Ⅰ~TM-Ⅶ). ICL, intracellular loop; ECL, extracellular loop; accession number: bovine GHSR1a, NP_001137208.1; human GHSR1a, NP_940799.1; pig GHSR1a, NP_999345.1; mouse GHSR1a, NP_796304.1; rat GHSR1a, NP_114464.1; Chinese hamster GHSR1a; XP_003499840.1.

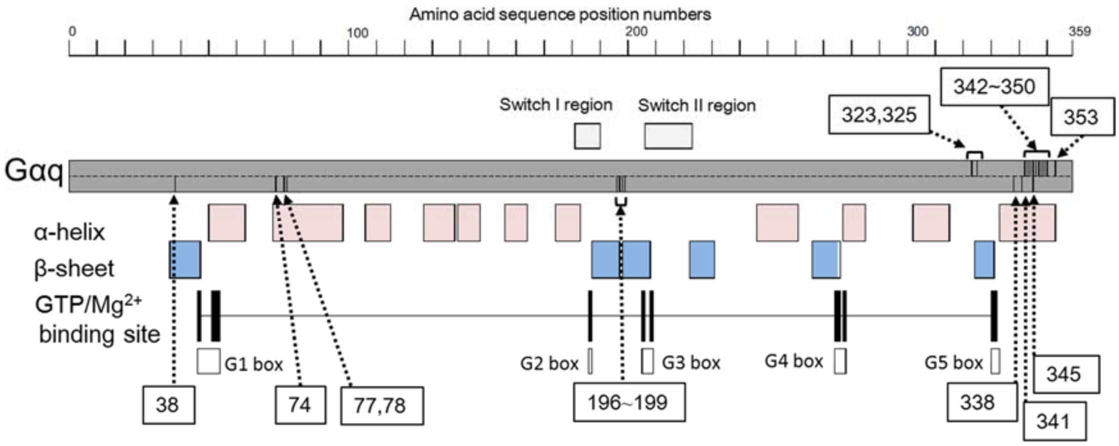

Figure S2 Structure of the bovine Gαq subunit and amino acid residues within 6 Å from the non-hydrogen atoms of the 4R region of the GHSR1a monomer. Pink boxes, α-helix; blue boxes, β-sheet; black boxes, GTP/Mg2+ binding site; gray boxes, switch I and II region, respectively. The reference sequence and structure of the Bovine Gαq are available at NCBI under accession number NP_001103472, and PDB under accession number 2BCJ_Q. In this model, residues 323, 325, 342–350, and 353 of Gαq were within 6 Å from the non-hydrogen atoms of the 4R region (residues 241–244) of one protomer, whereas residues 38, 74, 77, 78, 196–199, 338, 341, and 345 of Gαq were within 6 Å from those of the other protomer.

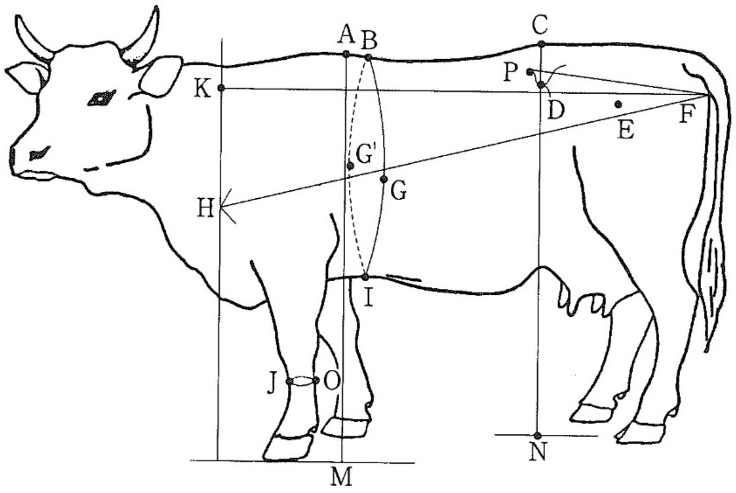

	Position	Name	Abbreviation
1	A - M	Withers height	WH
2	C - N	Hip height	HH
3	H - F	Body length	BL
4	B - I	Chest depth	CD
5	G – G′	Chest width	CW
6	P - F	Rump length	RL
7	D – D′	Hip width	HW
8	E – E′	Thurl width	TW
9	F – F′	Pin bone width	PBW
10	BGIG′B	Chest girth	CG
11	JOJ	Cannon circumference	CC

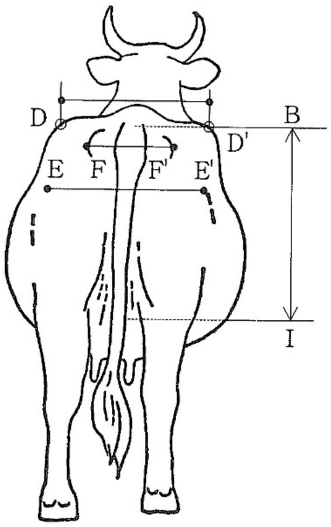

Figure S3 The position of 11 body shape and conformation measurements traits (Japanese Society of Animal Science, 2001 *Animal Husbandry Terminology Dictionary* (New Version). YOHKENDO Inc. Tokyo, Japan) .

Table S1 Primers used for DNA amplification, fragment analyses and sequencing (Komatsu *et al* . 2010)

Loci	Description	Primer Sequence (F, forward; R, reverse)	Amplified length (bp)
DelR242 (a 3-bp indel)			
	Fragment analysis and sequencing	F: 5'-TGTTTTCTGCCTCACTGTGC-3'<br>R: 5'-AAGACTCCGGGAGAGGAGAG-3'	168 bp (*4R allele*) and/or 165 bp (*3R* allele)
SNPs			
(1) *nt-7(C>A)* and (2) *nt70(C>G)*			
1st PCR	PCR	F: 5'-CTTTCCAAGCATCCTCCCTGAG-3'<br>R: 5'-GAAGCAGATGGCGAAGTAGCG-3'	584 bp
2nd PCR	Sequencing and/or PCR-RFLP	F: 5'-CAGTCGCGTCCCTGAACC-3'<br>R: 5'-CACGCAGGTGGCTGTGAC-3'	191 bp
(3) *nt456(G>A)*, (4) *nt580(G>A)* and (5) *nt667(C>T)*			
	Sequencing	F: 5'-TGGCCTTCTCCGACTTACTCA-3'<br>R: 5'-CAACTTCCTGCCGATGAGG-3'	468 bp
5' UTR microsatellite (*5'UTR-(TG)$_n$*)			
	Fragment analysis and sequencing	F: 5'-GGGCTGTGGGTCACTCTGTCC-3'<br>R: 5'-GAGGATGCTTGGAAAGGAAA-3'	~ 180 bp
Intron 1 microsatellite (*MS(GTTT)$_{5>6}$*)			
	Fragment analysis and sequencing	F: 5'-TGTGTTCACAGCCAAGTGAAA-3'<br>R: 5'-TGAGGAGCACCAGCAGATTT-3'	288 bp or 292 bp

Table S2 Allele frequencies of the *GHSR1a-DelR242*, *nt-7(C>A)*, *nt456 (G>A)*, *5'UTR microsatellite* (*5'UTR-(TG)$_n$*) loci and haplotype frequencies of the [*nt-7(C>A)*]-[*DelR242*], [*nt456(G>A)*]-[*DelR242*], [*nt-7(C>A)*]-[*nt456(G>A)*] and [*5'UTR-(TG)$_n$*]-[*nt-7(C>A)*]-[*DelR242*] in 95 sires, 17sires of 540 half sibs, 540 half sibs and 540 dam haploids of 540 half sibs in Japanese Shorthorn cattle.

Locus Allele or haplotype	95 sires (1)	17 sires of 540 half sibs (2)	540 half sibs (3)	540 dam haplotypes (4)
	(190)[a]	(34)[b]	(1080)[c]	(540)[d]
DelR242				
4R	0.595	0.412	0.462	0.686
3R	0.405	0.588	0.538	0.314
nt-7(C>A)				
A	0.732	0.735	0.718	0.806
C	0.268	0.265	0.282	0.194
nt456 (G>A)				
A	0.41	0.588	0.469	0.327
G	0.59	0.412	0.531	0.673
5'UTR-(TG)n				
19-TG[f]	0.016		0.008	0.017
20-TG	0.195	0.147	0.198	0.264
21-TG	0.011		0.006	0.013
22-TG	0.116	0.088	0.153	0.201
23-TG	0.211	0.206	0.207	0.156
24-TG	0.068	0.088	0.067	0.045
25-TG	0.153	0.088	0.100	0.135
26-TG	0.037		0.013	0.026
28-TG	0.179	0.382	0.244	0.135
31-TG	0.016		0.004	0.007
[*nt-7(C>A)*]-[*DelR242*]				
A-4R	0.495	0.294	0.440	0.608
C-4R	0.095	0.118	0.098	0.078
A-3R	0.237	0.382	0.274	0.198
C-3R	0.174	0.206	0.188	0.116
[*nt456(G>A)*]-[*DelR242*]				
A-4R			0.006	0.013
G-4R	0.589	0.412	0.531	0.673

A-3R	0.411	0.588	0.462	0.314
[*nt-7(C>A)*]-[*nt456(G>A)*]				
A-A	0.237	0.382	0.281	0.183
C-A	0.174	0.206	0.188	0.152
A-G	0.494	0.294	0.433	0.600
C-G	0.095	0.118	0.098	0.065
[*5'UTR-(TG)n*]-[*nt-7(C>A)*]-[*DelR242*]				
[19-TG]-A-4R	0.016		0.006	0.013
[19-TG]-A-3R			0.002	0.004
[20-TG]-A-4R	0.189	0.147	0.195	0.258
[20-TG]-A-3R			0.002	0.004
[20-TG]-C-4R	0.005		0.001	0.002
[21-TG]-A-4R	0.011		0.006	0.013
[22-TG]-A-4R	0.089	0.059	0.127	0.180
[22-TG]-A-3R			0.002	0.004
[22-TG]-C-4R	0.026	0.029	0.024	0.018
[23-TG]-A-3R	0.032		0.015	0.031
[23-TG]-C-4R	0.005		0.006	0.011
[23-TG]-C-3R	0.174	0.206	0.187	0.114
[24-TG]-A-4R	0.011			
[24-TG]-C-4R	0.058	0.088	0.067	0.045
[25-TG]-A-4R	0.153	0.088	0.098	0.131
[25-TG]-A-3R			0.001	0.002
[25-TG]-C-4R			0.001	0.002
[26-TG]-A-4R	0.026		0.007	0.013
[26-TG]-A-3R	0.011		0.001	0.013
[28-TG]-A-3R	0.179	0.382	0.244	0.135
[31-TG]-A-3R	0.016		0.003	0.006
[31-TG]-C-3R			0.001	0.002

Locus Allele or haplotype	Frequency differences								
	95 sire (1) vs. 17 sires (2)			95 sires (1) vs. 540 half sibs (3)			95 sires (1) vs. 540 dam haplotypes (4)		
	χ^2	d.f.[e]	*P* - value	χ^2	d.f.	*P* - value	χ^2	d.f.	*P* - value
DelR242	3.9	1	< 0.05	11.4	1	< 0.01	5.2	1	< 0.03

nt-7(C>A)	0.0	1		0.16	1		4.6	1	< 0.05
nt456 (G>A)	3.7	1		2.3	1		4.3	1	< 0.05
5'UTR-(TG)n	10.1	9		14.9	9	< 0.10	15.2	9	< 0.10
19-TG[f]	0.6	1		1.1	1		0.0	1	
20-TG	0.4	1		0.0	1		3.6	1	
21-TG	0.4	1		0.6	1		0.1	1	
22-TG	0.2	1		1.8	1		6.9	1	< 0.01
23-TG	0.0	1		0.0	1		3	1	
24-TG	0.2	1		0.0	1		1.5	1	
25-TG	1.0	1		4.7	1	< 0.03	0.4	1	
26-TG	1.3	1		5.7	1	< 0.03	0.6	1	
28-TG	7.2	1	< 0.01	3.8	1	< 0.10	2.2	1	
31-TG	0.6	1		4.0	1	< 0.05	1.2	1	
[*nt-7(C>A)*]-[*DelR242*]	5.2	3		2.1	3		8.2	3	< 0.05
A-4R	4.7	1	< 0.03	2.0	1		7.4	1	< 0.01
C-4R	0.2	1		0.0	1		0.5	1	
A-3R	3.2	1		1.1	1		1.3	1	
C-3R	0.2	1		0.2	1		4.2	1	< 0.05
[*nt456(G>A)*]-[*DelR242*]	3.7	1		3.1	2		7.9	2	< 0.02
A-4R				1.1	1		2.5	1	
G-4R	3.7	1		2.2	1		4.4	1	< 0.05
A-3R	3.7	1		1.7	1		5.9	1	< 0.02
[*nt-7(C>A)*]-[*nt456(G>A)*]	5.1	3		2.7	3		7.0	3	<0.10
A-A	3.2	1		1.6	1		2.6	1	
C-A	0.2	1		0.2	1		0.5	1	
A-G	4.6	1	< 0.05	2.4	1		6.5	1	< 0.02
C-G	0.2	1		0.0	1		1.9	1	
[*5'UTR-(TG)n*]-[*nt-7(C>A)*]-[*DelR242*]	69.8	15	< 0.001	41.3	21	< 0.01	30.7	21	< 0.10
[19-TG]-A-4R	6.5	1	< 0.03	2.0	1		0.1	1	
[19-TG]-A-3R		1		0.5	1		0.7	1	
[20-TG]-A-4R	1.3	1		0.0	1		3.6	1	

[20-TG]-A-3R		1		0.5	1		0.7	1	
[20-TG]-C-4R	2.2	1		1.5	1		0.6	1	
[21-TG]-A-4R	4.3	1	< 0.05	0.5	1		0.1	1	
[22-TG]-A-4R	1.5	1		2.4	1		8.7	1	< 0.01
[22-TG]-A-3R		1		0.5	1		0.7	1	
[22-TG]-C-4R	0.0	1		0.0	1		0.5	1	
[23-TG]-A-3R	12.9	1	< 0.01	2.6	1		0	1	
[23-TG]-C-4R	2.2	1		0.0	1		0.5	1	
[23-TG]-C-3R	0.6	1		0.2	1		4.5	1	< 0.05
[24-TG]-A-4R	4.3	1	< 0.05	7.2	1	< 0.01	5.7	1	< 0.02
[24-TG]-C-4R	1.2	1		0.2	1		0.5	1	
[25-TG]-A-4R	4.4	1	< 0.05	5.3	1	< 0.03	0.5	1	
[25-TG]-A-3R		1		0.2	1		0.4	1	
[25-TG]-C-4R		1		0.2	1		0.4	1	
[26-TG]-A-4R	10.8	1	< 0.01	5.6	1	< 0.02	1.6	1	
[26-TG]-A-3R	4.3	1	< 0.05	4.6	1	< 0.05	0.1	1	
[28-TG]-A-3R	14.8	1	< 0.01	4.2	1	< 0.05	2.2	1	
[31-TG]-A-3R	6.5	1	< 0.02	4.6	1	< 0.05	1.8	1	
[31-TG]-C-3R	1.0	1		0.4	1		0.4	1	

Locus<br>Allele or haplotype	Frequency differences								
	17 sires (2) vs.<br>540 half sibs (3)			17 sires (2) vs.<br>540 dam haplotypes (4)			540 half sibs (3) vs.<br>540 dam haplotypes (4)		
	χ^2	d.f.	*P* - value	χ^2	d.f.	*P* - value	χ^2	d.f.	*P* - value
DelR242	0.3	1		10.9	1	< 0.01	72.6	1	< 0.01
nt-7(C>A)	0.05	1		1.0	1		14.8	1	< 0.01
nt456 (G>A)	1.9	1		9.7	1	< 0.01	29.8	1	< 0.01
5'UTR-(TG)n	6.2	9		21.9	9	< 0.01	50.1	9	< 0.01
19-TG[f]	0.3	1		0.6	1		2.7	1	
20-TG	0.5	1		2.3	1		9.1	1	< 0.01
21-TG	0.1	1		0.0	1		2.1	1	
22-TG	1.1	1		2.6	1		5.9	1	< 0.02
23-TG	0.0	1		0.6	1		6.1	1	< 0.02
24-TG	0.2	1		1.3	1		3.1	1	
25-TG	0.1	1		0.6	1		8.6	1	< 0.01

26-TG	0.5	1		0.9	1		3.6	1	
28-TG	3.4	1		15.3	1	< 0.01	26.0	1	<0.01
31-TG	0.1	1		0.2	1		0.7	1	
[*nt-7(C>A)*]-[*DelR242*]	3.2	3		13.3	3	< 0.01	41.8	3	< 0.01
A-4R	2.9	1		13.0	1	< 0.01	40.6	1	< 0.01
C-4R	0.2	1		0.7	1		1.7	1	
A-3R	1.9	1		6.6	1	< 0.02	11.1	1	< 0.01
C-3R	0.1	1		2.4	1		13.6	1	< 0.01
[*nt456(G>A)*]-[*DelR242*]	2.2	2		11.0	2	< 0.01	33.8	2	< 0.01
A-4R	0.2	1		0.5	1		2.1	1	
G-4R	1.9	1		9.7	1	< 0.01	29.8	1	< 0.01
A-3R	2.1	1		10.9	1	< 0.01	32.5	1	< 0.01
[*nt-7(C>A)*]-[*nt456(G>A)*]	2.9	3		13.6	3	< 0.01	33.8	3	< 0.01
A-A	1.7	1		8.1	1	< 0.01	18.5	1	< 0.01
C-A	0.1	1		0.7	1		3.2	1	
A-G	2.6	1		12.3	1	< 0.01	40.2	1	< 0.01
C-G	0.2	1		1.4	1		4.9	1	< 0.03
[*5'UTR-(TG)n*]-[*nt-7(C>A)*]-[*DelR242*]	18.4	20		40.0	20	< 0.01	131.9	20	< 0.001
[19-TG]-A-4R	0.8	1		1.0	1		2.8	1	
[19-TG]-A-3R	0.3	1		0.3	1		0.6	1	
[20-TG]-A-4R	1.3	1		3.5	1		12.3	1	< 0.01
[20-TG]-A-3R	0.3	1		0.3	1		0.6	1	
[20-TG]-C-4R	0.2	1		0.2	1		0.3	1	
[21-TG]-A-4R	0.8	1		1.0	1		2.8	1	
[22-TG]-A-4R	4.1	1	< 0.05	6.0	1	< 0.02	11.7	1	< 0.01
[22-TG]-A-3R	0.3	1		0.3	1		0.6	1	
[22-TG]-C-4R	0.1	1		0.4	1		1.4	1	
[23-TG]-A-3R	1.9	1		2.4	1		6.4	1	< 0.02
[23-TG]-C-4R	0.8	1		0.9	1		1.7	1	
[23-TG]-C-3R	0.2	1		3.7	1		27.6	1	< 0.01
[24-TG]-A-4R									

[24-TG]-C-4R	0.6	1		1.9	1		6.0	1	< 0.02
[25-TG]-A-4R	0.1	1		1.0	1		6.2	1	< 0.02
[25-TG]-A-3R	0.2	1		0.2	1		0.3	1	
[25-TG]-C-4R	0.2	1		0.2	1		0.3	1	
[26-TG]-A-4R	0.9	1		1.0	1		2.0	1	
[26-TG]-A-3R	0.2	1		1.0	1		9.2	1	< 0.01
[28-TG]-A-3R	7.1	1	< 0.01	19.2	1	< 0.01	50.9	1	< 0.01
[31-TG]-A-3R	0.4	1		0.5	1		0.9	1	
[31-TG]-C-3R	0.9	1		0.5	1		0.0	1	

[a]A total of 190 haplotypes derived from 95 sires.

[b]A total of 34 haplotypes derived from 17 sires of 540 half sibs. The sire genotypes were 14 *4R/3R* heterozygotes and three *3R/3R* homozygotes.

[c]A total number of 1080 haplotypes derived from 540 half sibs.

[d]A total number of 540 haplotypes derived from 540 dams of 540 half sibs. A dam's allele or haplotype was estimated by removing the sire's transmitted allele or haplotype from the half sib genotypes or haplotype combinations. The sire genotypes were 14 *4R/3R* heterozygotes and 3 *3R/3R* homozygotes.

[e]Degrees of freedom.

[f]$(TG)_{19}$.

Table S3 Comparison of relative growth rates (RGR) of body shape and conformation traits among the *DelR242* genotypes in direct-tested weaner bulls.

Traits[a,b]	No. of animals	Mean (SD)[c] [%/month]	Genotype mean (SD) [%/month]			*P* - value[d]
			4R/4R	*4R/3R*	*3R/3R*	
BW[a]_RGR[b]	121	9.27	9.02[ab]	9.56[a]	8.90[b]	0.0085
		(0.85)	(0.84)	(0.83)	(0.80)	(*)
WH_RGR	121	2.11	2.05	2.11	2.21	0.3088
		(0.30)	(0.28)	(0.34)	(0.24)	(ns)
CW_RGR	94	4.13	3.16[c]	4.73[a]	3.96[ac]	0.0033
		(1.49)	(1.32)	(1.62)	(1.14)	(*)
RL_RGR	94	3.01	2.75	2.99	3.34	0.2306
		(0.98)	(0.90)	(1.04)	(0.93)	(ns)
CC_RGR	94	2.48	2.63	2.30	2.71	0.1528
		(0.75)	(0.85)	(0.63)	(0.80)	(ns)
No. of weaner bulls	121		33	61	27	
	94		25	47	22	

[a]Traits: BW, Body weight; WH, Withers height; CW, Chest width; CD, Chest depth; BL, Body length; RL, Rump length; HW, Hip width; TW, Thurl width; CG, Chest girth; CC, Cannon circumference in direct-tested weaner bulls

[b]Relative growth rate (RGR) [%/month] = {[(BMt2 - BMt1)/(t2 - t1)] / [(BMt2 + BMt1)/2]} × 100 × 30, where BMt1 or BMt2 denotes body measurement (BM) at the start of direct-testing (t1; days) or at the end of direct-testing (t2, days).

[c]SD, standard deviation.

[d]*P* - valued (Bonferroni correction), $P = 0.10/5 = 0.02$; $P = 0.05/5 = 0.01$; $P = 0.01/5 = 0.0025$. a,b: $P < 0.05/3 = 0.017$; a,c: $P < 0.01/3 = 0.003$.

Table S4 Changes in the ratio of the *4R/3R* heterozygous individual in his progenies produced by mating between the progeny-tested sire and dams depending on the *3R* allele frequency in the dam population.

Allele frequency in the dam population		Genotype of the progeny-tested sire		
		4R/4R	*4R/3R*	*3R/3R*
4R	*3R*	Ratio of the *4R/3R* individual in his progenies (%)		
0.9	0.1	10	50	90
0.8	0.2	20	50	80
0.7	0.3	30	50	70
0.6	0.4	40	50	60
0.5	0.5	50	50	50
0.4	0.6	60	50	40
0.3	0.7	70	50	30
0.2	0.8	80	50	20
0.1	0.9	90	50	10

Table S5 Summary statistics of growth, feed intake, body shape and conformation measurements traits in direct-tested bulls, carcass traits in shipped half-sibs, additive and dominance effects of the *C* allele of the *nt-7(C>A)* locus and epistatic effect between the *nt-7(C>A)* and *DelR242* loci.

Traits (abbreviation)[a](units)	No. of animals	Least squares mean						*P* -value[e]	Effect of the *C* allele				Epistatic effect[g]	
		A/A	(SD)[c]	*A/C*	(SD)	*C/C*	(SD)		LRT[f]	*P* - value	Addi - tive effect	Domi - nant effect	LRT	*P* - value
(1) Direct-tested bulls		(58)[d]		(41)		(22)								
AGE (day)	121	242.50	(20.7)	250.90	(20.5)	241.23	(19.2)	ns						
ADG (kg/day)	121	1.30	(0.14)	1.27	(0.15)	1.26	(0.15)	0.113[ns]	4.36	0.113	-0.03	-0.01	0.20	ns
180BW (kg)	121	244.40	(32.0)	235.60	(28.0)	248.70	(31.7)	0.562[ns]	1.153	0.562	1.53	-5.36	0.52	ns
365BW (kg)	121	486.7	(36.0)	470.5	(32.7)	482.9	(47.2)	0.228[ns]	2.959	0.228	-4.24	-8.29	0.04	ns
8SFT (mm)	121	79.7	(15.5)	81.5	(16.6)	77.7	(14.7)	0.023[ns]	7.56	0.023	0.00	5.24	0.39	ns
ROUGH (%)	121	45.0	(2.4)	44.8	(2.3)	45.2	(1.9)	0.776[ns]	0.51	0.776	0.15	-0.01	1.88	ns
ROUGH_1 (kg))	121	3.75	(0.48)	3.83	(0.52)	3.89	(0.48)	0.179[ns]	3.44	0.179	0.10	-0.02	0.09	ns
CONC_1 (kg)	121	4.50	(0.52)	4.61	(0.58)	4.63	(0.55)	0.360[ns]	2.04	0.360	0.08	-0.03	0.39	ns
Body shape and conformation measurements:														
WH_S (cm)	121	115.3	(3.4)	115.3	(3.5)	114.7	(3.9)	0.272[ns]	2.61	0.272	-0.49	-0.17	3.56	ns

WH_E (cm)	121	127.1	(3.1)	127.5	(3.4)	126.6	(3.7)	0.570^{ns}	1.17	0.570	-0.28	0.48	1.92	ns
		(40)		(34)		(20)								
CW_S (cm)	94	38.4	(3.4)	38.6	(4.0)	39.4	(4.0)	0.829^{ns}	0.37	0.829	0.07	-0.35	0.40	ns
CW_E (cm)	94	46.7	(2.8)	46.7	(3.2)	46.0	(2.7)	0.796^{ns}	0.46	0.796	-0.23	-0.08	0.81	ns
RL_S (cm)	94	42.7	(2.2)	42.8	(2.3)	41.9	(2.5)	0.496^{ns}	1.40	0.496	-0.28	0.01	0.05	ns
RL_E (cm)	94	48.8	(2.1)	48.9	(2.5)	49.3	(1.9)	0.412^{ns}	1.77	0.412	0.17	-0.50	1.05	ns
CC_S (cm)	94	17.7	(0.8)	17.6	(0.7)	17.6	(0.8)	0.576^{ns}	1.10	0.576	-0.11	-0.01	0.13	ns
CC_E (cm)	94	19.9	(0.6)	19.9	(0.7)	19.7	(0.9)	0.934^{ns}	0.14	0.934	-0.03	0.03	0.87	ns
(2) Shipped half sibs[b]		(282)		(208)		(47)								
Slaughter age (month)		24.2	(3.2)	24.0	(3.3)	24.6	(3.5)	—						
CW (kg)	537	405.0	(38.1)	409.6	(37.2)	407.0	(41.3)	0.282^{ns}	2.53	0.282	-0.57	4.97	0.83	ns
LMA (cm^2)	537	47.9	(5.2)	48.2	(5.3)	48.6	(5.4)	0.989^{ns}	0.02	0.989	0.06	0.00	0.54	ns
RT (cm)	537	6.37	(0.66)	6.39	(0.62)	6.38	(0.73)	0.646^{ns}	0.87	0.646	0.01	0.05	1.03	ns
SFT (cm)	537	2.35	(0.64)	2.43	(0.66)	2.42	(0.69)	0.093^{ns}	4.76	0.093	0.03	0.10	0.04	ns
BMS	537	2.11	(0.36)	2.12	(0.37)	2.04	(0.20)	0.422^{ns}	1.72	0.422	-0.02	0.05	0.01	ns
Firmness	537	2.09	(0.30)	2.09	(0.28)	2.04	(0.20)	0.512^{ns}	1.34	0.512	-0.03	0.02	0.11	ns

Texture	537	2.39	(0.50)	2.37	(0.48)	2.21	(0.41)	0.210[ns]	3.12	0.210	-0.06	0.08	0.65	ns

[a]AGE, age at the start of direct-testing; ADG, average dairy gain; 180BW, 180-day adjusted body weight; 365BW, 365-day adjusted body weight; 8SFT, total thickness of eight points of subcutaneous fat; ROUGH, roughage intake; ROUGH_1, roughage weights per kilogram body weight gain; CONC_1, concentration weights per kilogram body weight gain; WH_S, withers height at the start of direct-testing; WH_E, withers height at the end of direct-testing; CW_S, chest width at the start of direct-testing; CW_E, chest width at the end of direct-testing; RL_S, rump length at the start of direct-testing; RL_E, rump length at the end of direct-testing; CC_S, cannon circumference at the start of direct-testing; CC_E, cannon circumference at the end of direct-testing; CW, carcass weight; LMA, longissimus muscle area; RT, rib thickness; SFT, subcutaneous fat thickness; BMS, beef marbling score.

[b]209 dams and 328 steers. [c]Standard deviation. [d]Number of animals. [e]*P* - value [(Significance level (Bonferroni correction): ns, not significant; **P* = 0.05/15 = 0.0033; 15, a number of traits for direct-tested bulls)]. [f]Likelihood ratio test. [g]Epistatic effect estimated by the epi_snp option between the *DelR242* and *nt-7(C>A)* loci .
