# Supplementary material for: Overdominance Effect of the Bovine Ghrelin Receptor (GHSR1a)-DelR242 Locus on Growth in Japanese Shorthorn Weaner Bulls: Heterozygote Advantage in Bull Selection and Molecular Mechanisms
Source: G3 (Bethesda). 2014 Dec 23;5(2):271–9. doi: 10.1534/g3.114.016105 (PMC4321035; doi:10.1534/g3.114.016105)
Supplement: Supporting Information [file supp_g3.114.016105_FileS1.pdf]

File S1

Materials and Methods: Modeling of the GHSR1a-Gαq complex structure.

Although the structure of the complex between a GPCR dimer and a G protein has not yet been determined experimentally, a crystal structure of the complex between a GPCR monomer and a G protein is available. Therefore, we took a two-step approach to the modeling of the GHSR1a-Gαq complex structure, where the structures of the GHSR1a monomer and the GHSR1a monomer-Gαq complex were first modeled, and then the GHSR1a monomer model structure was docked to the GHSR1a monomer-Gαq complex structure. The structure of the 4R type of the bovine GHSR1a monomer and that of the complex between the 4R type of bovine GHSR1a and Gαq were predicted by comparative modeling. The structure of bovine inactive rhodopsin (PDB ID: 2I35) was used as the template for modeling the GHSR1a monomer, whereas the structures of active opsin (PDB ID: 3CAP) and the complex of bovine β2 adrenergic receptor¬ and Gs (PDB ID: 3SN6) were used as the templates for modeling the GHSR1a-Gαq complex. The sequence alignments between GHSR1a (GenBank Protein ID: DAA33245.1) and rhodopsin, opsin or β2 adrenergic receptor were obtained using the AlignMe server, which considers the transmembrane region for sequence alignment (Stamm *et al*. 2013). The sequence of Gαq (Refseq ID: NP_001103472.1) was aligned to that of Gs with the BLOSUM62 matrix (Henikoff and Henikoff 1992), and a gap opening penalty of 12 and gap extension penalty of 1 were incorporated using a sequence-alignment tool of UCSF Chimera (Meng *et al*. 2006). The structural models were generated using the program Modeller (Sali and Blundell 1993). Next, docking models between the GHSR1a monomer and GHSR1a-Gαq complex were generated by the ZDOCK 3.0.2 program (Pierce *et al*. 2011). The tilt angles of the GHSR1a monomer with respect to the normal membrane surface and the offset along the normal of the center-of-mass position of the GHSR1a monomer from that of the GHSR1a in the GHSR1a-Gαq complex were calculated for each representative structure using the FiPD program (Casciari *et al*. 2006). We selected the models with tilt angles and offsets of less than 0.5 radians and 5.0 Å, respectively. The interactions within the model structures were examined to identify the dimer interface between the GHSR1a protomers. The model having the best ZDOCK score was selected as the final model.

REFERENCE

Casciari, D., M. Seeber, and F. Fanelli, 2006 Quaternary structure predictions of transmembrane proteins starting from the monomer: a docking-based approach. BMC Bioinformatics 7: 340.

Henikoff, S., and J. G. Henikoff, 1992 Amino acid substitution matrices from protein blocks. Proc. Natl. Acad. Sci. USA. 89: 10915-10919.

Meng, E. C., E. F. Pettersen, G. S. Couch, C. C. Huang, and T. E. Ferrin, 2006 Tools for integrated sequence-structure analysis with UCSF Chimera. BMC Bioinformatics 7: 339.

Pierce, B.G., Y. Hourai, and Z. Weng, 2011 Accelerating protein docking in ZDOCK using an advanced 3D convolution library. PLoS One 6: e24657.

Sali, A., and T. L. Blundell, 1993 Comparative protein modelling by satisfaction of spatial restraints. J. Mol. Biol. 234: 779-815.

Stamm, M., R. Staritzbichler, K. Khafizov, and L. R. Forrest, 2013 Alignment of helical membrane protein sequences using AlignMe. PLoS One 8: e57731.
