# Supplementary material for: Overdominance Effect of the Bovine Ghrelin Receptor (GHSR1a)-DelR242 Locus on Growth in Japanese Shorthorn Weaner Bulls: Heterozygote Advantage in Bull Selection and Molecular Mechanisms
Source: G3 (Bethesda). 2014 Dec 23;5(2):271–9. doi: 10.1534/g3.114.016105 (PMC4321035; doi:10.1534/g3.114.016105)
Supplement: Supporting Information [file supp_g3.114.016105_FileS2.pdf]

File S2

Materials and Methods: Direct-testing and progeny-testing programs in Japanese Shorthorn bulls.

(1) Direct-testing program**:** The calves were born between February and May in their respective farmers' feedlots, and grazed in regional public ranches with their dams from May to the end of September. The preliminary selection based on their body shape and conformation measurements (BSCM) was carried out at 4~5 months of age (Figure S3). 15 weaner bulls at 6–7 months of age were selected from a pool of 350 weaner bulls and transported to the direct-testing station at the Animal Industry Research Institute, Iwate Agricultural Research Center (IARC). They were fed with concentrate and had *ad libitum* access to roughage with an allowance of 42 days for adjustment and acclimatization, followed by the standard 140 days of direct-testing prior to slaughter. The average age at the start of direct-testing was 245.1 ± 20.6 days (mean ± SD). Routine management of the animals involved fortnightly recording of body weight (BW) and BSCM traits at the start and end of direct-testing (see MATERIALS AND METHODS: Animals and data collection). 5 weaner bulls were selected from a group of 15 weaner bulls based on their selection indices for direct-testing, pedigree and BSCM data. The selection index formula used for direct-testing of Japanese Shorthorn bulls is:

[21.749 × ADG of direct-testing period] – [0.254 × 8SFT] + 10.

(2) Progeny-testing program**:** 5 selected young bulls were mated with ordinary cows and their progeny (more than 6 half sib steer calves) were performance-tested. They were allowed to suckle their dams in addition to being fed concentrates and timothy-grass hay until weaning. After weaning, they were moved to the grower's barn and reared until the attainment of 7–8 months of age. They were fed with the conventional grower ration with an allowance of 20 days for adjustment and acclimatization followed by the standard 308 days (44 weeks) progeny-testing duration prior to slaughter. Routine management of the animals involved the recording of body weight, BSCM traits, concentrate and roughage intakes every 4 weeks. Steers were weighed at the beginning and end (WT) of the testing period so that average daily gain (ADG) could be computed. Carcass data collected included slaughter weight (WS), cold carcass weight (CW), ADG, rib eye area (REA), rib thickness (RT), carcass yield estimate (YE), subcutaneous fat thickness (SFT), inter-muscular fat thickness (IFT) and beef marbling score (BMS). One bull was selected from 5 bulls based on their aggregate breeding value, pedigree and BSCM data for progeny-testing. The aggregate breeding value formula used for progeny-testing of directly-tested bulls is:

[11.1 × breeding value of ADG at direct-testing] – [0.031 × breeding value of 8SFT at direct-testing] + [4.67 × breeding value of ADG at progeny-testing] + [0.025 × REA at progeny-testing] + [1.69 × breeding value of SFT at progeny-testing] + [0.807 × BMS at progeny-testing].
