# Supplementary material for: Overdominance Effect of the Bovine Ghrelin Receptor (GHSR1a)-DelR242 Locus on Growth in Japanese Shorthorn Weaner Bulls: Heterozygote Advantage in Bull Selection and Molecular Mechanisms
Source: G3 (Bethesda). 2014 Dec 23;5(2):271–9. doi: 10.1534/g3.114.016105 (PMC4321035; doi:10.1534/g3.114.016105)
Supplement: Supporting Information [file supp_g3.114.016105_FigureS1.pdf]

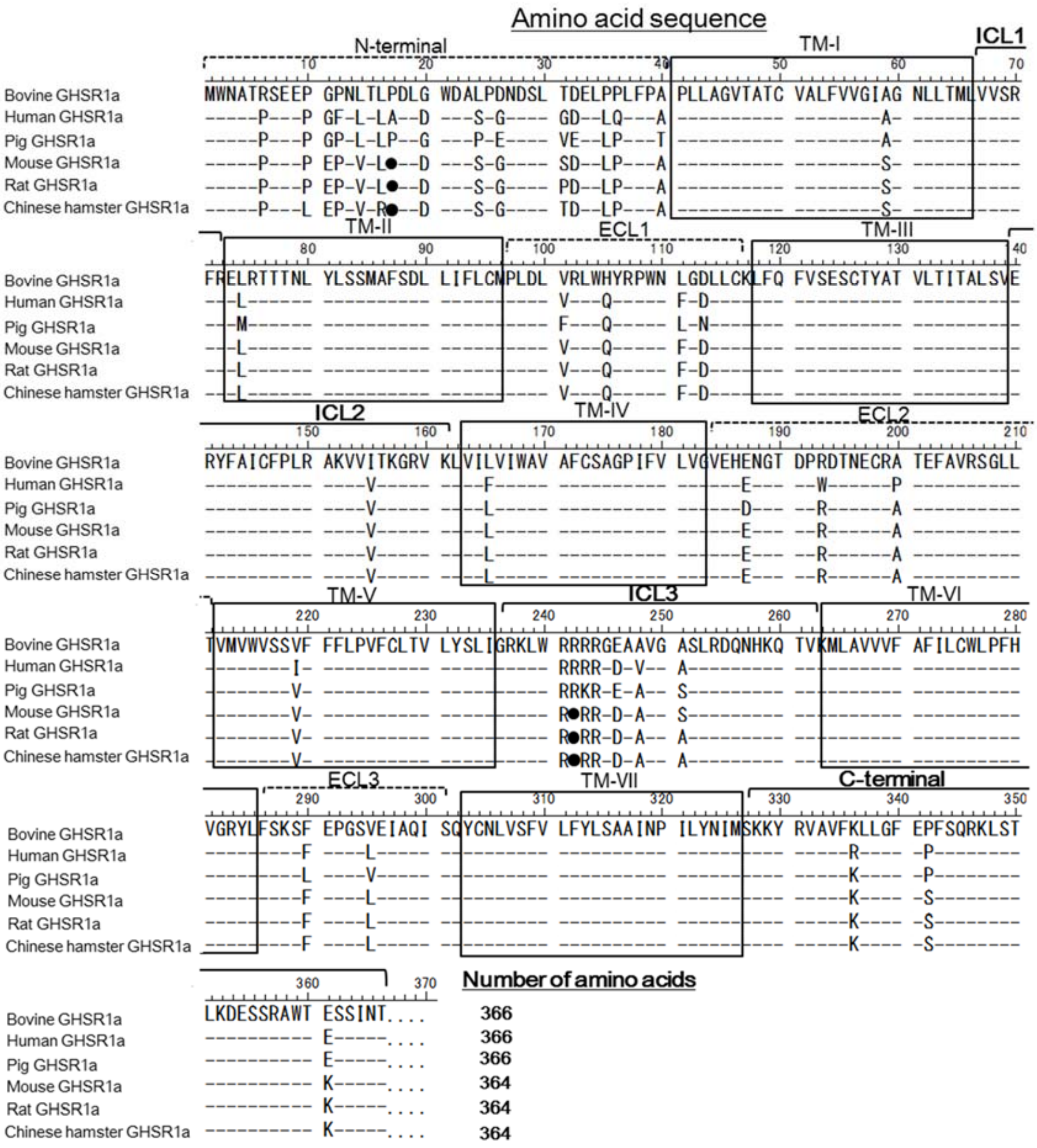

Figure S1 Differential amino acid sequences of the GHSR1a protein among six mammalian species. The dashes indicate identical residues. Black circles represent amino-acid deletions. The transmembrane helices are shown in boxes (TM-Ⅰ~TM-Ⅶ). ICL, intracellular loop; ECL, extracellular loop; accession number: bovine GHSR1a, NP_001137208.1; human GHSR1a, NP_940799.1; pig GHSR1a, NP_999345.1; mouse GHSR1a, NP_796304.1; rat GHSR1a, NP_114464.1; Chinese hamster GHSR1a; XP_003499840.1.
