# Supplementary material for: Overdominance Effect of the Bovine Ghrelin Receptor (GHSR1a)-DelR242 Locus on Growth in Japanese Shorthorn Weaner Bulls: Heterozygote Advantage in Bull Selection and Molecular Mechanisms
Source: G3 (Bethesda). 2014 Dec 23;5(2):271–9. doi: 10.1534/g3.114.016105 (PMC4321035; doi:10.1534/g3.114.016105)
Supplement: Supporting Information [file supp_g3.114.016105_FigureS2.pdf]

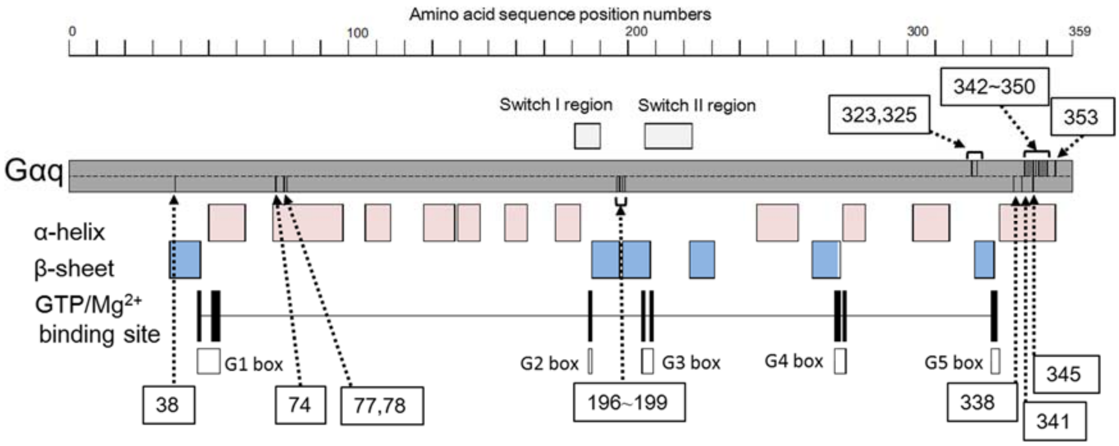

Figure S2 Structure of the bovine Gαq subunit and amino acid residues within 6 Å from the non-hydrogen atoms of the 4R region of the GHSR1a monomer. Pink boxes, α-helix; blue boxes, β-sheet; black boxes, GTP/Mg2+ binding site; gray boxes, switch I and II region, respectively. The reference sequence and structure of the Bovine Gαq are available at NCBI under accession number NP_001103472, and PDB under accession number 2BCJ_Q. In this model, residues 323, 325, 342–350, and 353 of Gαq were within 6 Å from the non-hydrogen atoms of the 4R region (residues 241–244) of one protomer, whereas residues 38, 74, 77, 78, 196–199, 338, 341, and 345 of Gαq were within 6 Å from those of the other protomer.
