# Supplementary material for: Overdominance Effect of the Bovine Ghrelin Receptor (GHSR1a)-DelR242 Locus on Growth in Japanese Shorthorn Weaner Bulls: Heterozygote Advantage in Bull Selection and Molecular Mechanisms
Source: G3 (Bethesda). 2014 Dec 23;5(2):271–9. doi: 10.1534/g3.114.016105 (PMC4321035; doi:10.1534/g3.114.016105)
Supplement: Supporting Information [file supp_g3.114.016105_FigureS3.pdf]

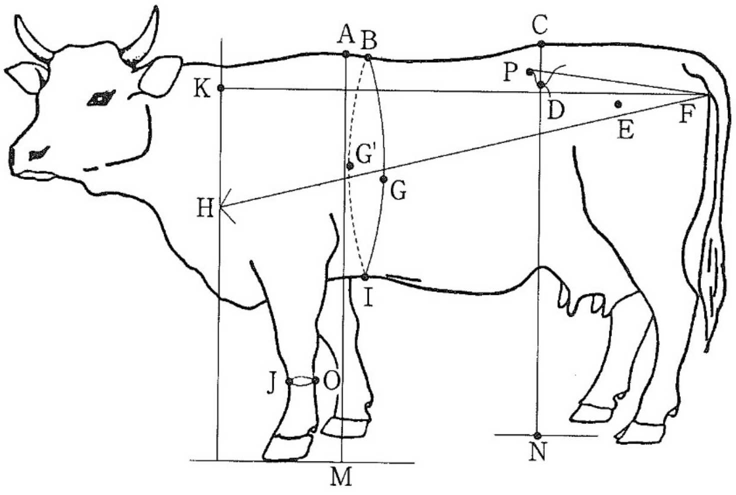

	Position	Name	Abbreviation
1	A - M	Withers height	WH
2	C - N	Hip height	HH
3	H - F	Body length	BL
4	B - I	Chest depth	CD
5	G – G′	Chest width	CW
6	P - F	Rump length	RL
7	D – D′	Hip width	HW
8	E – E′	Thurl width	TW
9	F – F′	Pin bone width	PBW
10	BGIG′B	Chest girth	CG
11	JOJ	Cannon circumference	CC

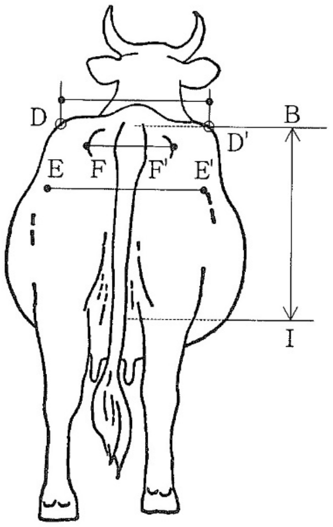

Figure S3 The position of 11 body shape and conformation measurements traits (Japanese Society of Animal Science, 2001 *Animal Husbandry Terminology Dictionary* (New Version). YOHKENDO Inc. Tokyo, Japan) .
