# Supplementary material for: Overdominance Effect of the Bovine Ghrelin Receptor (GHSR1a)-DelR242 Locus on Growth in Japanese Shorthorn Weaner Bulls: Heterozygote Advantage in Bull Selection and Molecular Mechanisms
Source: G3 (Bethesda). 2014 Dec 23;5(2):271–9. doi: 10.1534/g3.114.016105 (PMC4321035; doi:10.1534/g3.114.016105)
Supplement: Supporting Information [file supp_g3.114.016105_TableS1.pdf]

**Table S1 Primers used for DNA amplification, fragment analyses and sequencing** (Komatsu *et al* . 2010)

| Loci | Description | Primer Sequence (F, forward; R, reverse) | Amplified length (bp) |
|---|---|---|---|
| *DelR242* (a 3-bp indel) | | | |
| | Fragment analysis and sequencing | F: 5'-TGTTTTCTGCCTCACTGTGC-3'<br>R: 5'-AAGACTCCGGGAGAGGAGAG-3' | 168 bp (*4R allele*) and/or 165 bp (*3R* allele) |
| SNPs | | | |
| (1) *nt-7(C>A)* and (2) *nt70(C>G)* | | | |
| 1st PCR | PCR | F: 5'-CTTTCCAAGCATCCTCCCTGAG-3'<br>R: 5'-GAAGCAGATGGCGAAGTAGCG-3' | 584 bp |
| 2nd PCR | Sequencing and/or PCR-RFLP | F: 5'-CAGTCGCGTCCCTGAACC-3'<br>R: 5'-CACGCAGGTGGCTGTGAC-3' | 191 bp |
| (3) *nt456(G>A)*, (4) *nt580(G>A)* and (5) *nt667(C>T)* | | | |
| | Sequencing | F: 5'-TGGCCTTCTCCGACTTACTCA-3'<br>R: 5'-CAACTTCCTGCCGATGAGG-3' | 468 bp |
| 5' UTR microsatellite (*5'UTR-(TG)$_n$*) | | | |
| | Fragment analysis and sequencing | F: 5'-GGGCTGTGGGTCACTCTGTCC-3'<br>R: 5'-GAGGATGCTTGGAAAGGAAA-3' | ~ 180 bp |
| Intron 1 microsatellite (*MS(GTTT)$_{5>6}$*) | | | |
| | Fragment analysis and sequencing | F: 5'-TGTGTTCACAGCCAAGTGAAA-3'<br>R: 5'-TGAGGAGCACCAGCAGATTT-3' | 288 bp or 292 bp |
