# Supplementary material for: Overdominance Effect of the Bovine Ghrelin Receptor (GHSR1a)-DelR242 Locus on Growth in Japanese Shorthorn Weaner Bulls: Heterozygote Advantage in Bull Selection and Molecular Mechanisms
Source: G3 (Bethesda). 2014 Dec 23;5(2):271–9. doi: 10.1534/g3.114.016105 (PMC4321035; doi:10.1534/g3.114.016105)
Supplement: Supporting Information [file supp_g3.114.016105_TableS2.pdf]

Table S2 Allele frequencies of the *GHSR1a-DelR242*, *nt-7(C>A)*, *nt456 (G>A)*, *5'UTR microsatellite* (*5'UTR-(TG)*$_{n}$) loci and haplotype frequencies of the [*nt-7(C>A)*]-[*DelR242*], [*nt456(G>A)*]-[*DelR242*], [*nt-7(C>A)*]-[*nt456(G>A)*] and [*5'UTR-(TG)*$_{n}$]-[*nt-7(C>A)*]-[*DelR242*] in 95 sires, 17sires of 540 half sibs, 540 half sibs and 540 dam haploids of 540 half sibs in Japanese Shorthorn cattle.

Locus Allele or haplotype	95 sires (1)	17 sires of 540 half sibs (2)	540 half sibs (3)	540 dam haplotypes (4)
	(190)[a]	(34)[b]	(1080)[c]	(540)[d]
DelR242				
4R	0.595	0.412	0.462	0.686
3R	0.405	0.588	0.538	0.314
nt-7(C>A)				
A	0.732	0.735	0.718	0.806
C	0.268	0.265	0.282	0.194
nt456 (G>A)				
A	0.41	0.588	0.469	0.327
G	0.59	0.412	0.531	0.673
5'UTR-(TG)n				
19-TG[f]	0.016		0.008	0.017
20-TG	0.195	0.147	0.198	0.264
21-TG	0.011		0.006	0.013
22-TG	0.116	0.088	0.153	0.201
23-TG	0.211	0.206	0.207	0.156
24-TG	0.068	0.088	0.067	0.045
25-TG	0.153	0.088	0.100	0.135
26-TG	0.037		0.013	0.026
28-TG	0.179	0.382	0.244	0.135
31-TG	0.016		0.004	0.007
[*nt-7(C>A)*]-[*DelR242*]				
A-4R	0.495	0.294	0.440	0.608
C-4R	0.095	0.118	0.098	0.078
A-3R	0.237	0.382	0.274	0.198
C-3R	0.174	0.206	0.188	0.116
[*nt456(G>A)*]-[*DelR242*]				
A-4R			0.006	0.013
G-4R	0.589	0.412	0.531	0.673

A-3R	0.411	0.588	0.462	0.314
[*nt-7(C>A)*]-[*nt456(G>A)*]				
A-A	0.237	0.382	0.281	0.183
C-A	0.174	0.206	0.188	0.152
A-G	0.494	0.294	0.433	0.600
C-G	0.095	0.118	0.098	0.065
[*5'UTR-(TG)n*]-[*nt-7(C>A)*]-[*DelR242*]				
[19-TG]-A-4R	0.016		0.006	0.013
[19-TG]-A-3R			0.002	0.004
[20-TG]-A-4R	0.189	0.147	0.195	0.258
[20-TG]-A-3R			0.002	0.004
[20-TG]-C-4R	0.005		0.001	0.002
[21-TG]-A-4R	0.011		0.006	0.013
[22-TG]-A-4R	0.089	0.059	0.127	0.180
[22-TG]-A-3R			0.002	0.004
[22-TG]-C-4R	0.026	0.029	0.024	0.018
[23-TG]-A-3R	0.032		0.015	0.031
[23-TG]-C-4R	0.005		0.006	0.011
[23-TG]-C-3R	0.174	0.206	0.187	0.114
[24-TG]-A-4R	0.011			
[24-TG]-C-4R	0.058	0.088	0.067	0.045
[25-TG]-A-4R	0.153	0.088	0.098	0.131
[25-TG]-A-3R			0.001	0.002
[25-TG]-C-4R			0.001	0.002
[26-TG]-A-4R	0.026		0.007	0.013
[26-TG]-A-3R	0.011		0.001	0.013
[28-TG]-A-3R	0.179	0.382	0.244	0.135
[31-TG]-A-3R	0.016		0.003	0.006
[31-TG]-C-3R			0.001	0.002

Locus Allele or haplotype	Frequency differences								
	95 sire (1) vs. 17 sires (2)			95 sires (1) vs. 540 half sibs (3)			95 sires (1) vs. 540 dam haplotypes (4)		
	χ^2	d.f.[e]	*P* - value	χ^2	d.f.	*P* - value	χ^2	d.f.	*P* - value
DelR242	3.9	1	< 0.05	11.4	1	< 0.01	5.2	1	< 0.03

nt-7(C>A)	0.0	1		0.16	1		4.6	1	< 0.05
nt456 (G>A)	3.7	1		2.3	1		4.3	1	< 0.05
5'UTR-(TG)n	10.1	9		14.9	9	< 0.10	15.2	9	< 0.10
19-TG[f]	0.6	1		1.1	1		0.0	1	
20-TG	0.4	1		0.0	1		3.6	1	
21-TG	0.4	1		0.6	1		0.1	1	
22-TG	0.2	1		1.8	1		6.9	1	< 0.01
23-TG	0.0	1		0.0	1		3	1	
24-TG	0.2	1		0.0	1		1.5	1	
25-TG	1.0	1		4.7	1	< 0.03	0.4	1	
26-TG	1.3	1		5.7	1	< 0.03	0.6	1	
28-TG	7.2	1	< 0.01	3.8	1	< 0.10	2.2	1	
31-TG	0.6	1		4.0	1	< 0.05	1.2	1	
[*nt-7(C>A)*]-[*DelR242*]	5.2	3		2.1	3		8.2	3	< 0.05
A-4R	4.7	1	< 0.03	2.0	1		7.4	1	< 0.01
C-4R	0.2	1		0.0	1		0.5	1	
A-3R	3.2	1		1.1	1		1.3	1	
C-3R	0.2	1		0.2	1		4.2	1	< 0.05
[*nt456(G>A)*]-[*DelR242*]	3.7	1		3.1	2		7.9	2	< 0.02
A-4R				1.1	1		2.5	1	
G-4R	3.7	1		2.2	1		4.4	1	< 0.05
A-3R	3.7	1		1.7	1		5.9	1	< 0.02
[*nt-7(C>A)*]-[*nt456(G>A)*]	5.1	3		2.7	3		7.0	3	<0.10
A-A	3.2	1		1.6	1		2.6	1	
C-A	0.2	1		0.2	1		0.5	1	
A-G	4.6	1	< 0.05	2.4	1		6.5	1	< 0.02
C-G	0.2	1		0.0	1		1.9	1	
[*5'UTR-(TG)n*]-[*nt-7(C>A)*]-[*DelR242*]	69.8	15	< 0.001	41.3	21	< 0.01	30.7	21	< 0.10
[19-TG]-A-4R	6.5	1	< 0.03	2.0	1		0.1	1	
[19-TG]-A-3R		1		0.5	1		0.7	1	
[20-TG]-A-4R	1.3	1		0.0	1		3.6	1	

[20-TG]-A-3R		1		0.5	1		0.7	1	
[20-TG]-C-4R	2.2	1		1.5	1		0.6	1	
[21-TG]-A-4R	4.3	1	< 0.05	0.5	1		0.1	1	
[22-TG]-A-4R	1.5	1		2.4	1		8.7	1	< 0.01
[22-TG]-A-3R		1		0.5	1		0.7	1	
[22-TG]-C-4R	0.0	1		0.0	1		0.5	1	
[23-TG]-A-3R	12.9	1	< 0.01	2.6	1		0	1	
[23-TG]-C-4R	2.2	1		0.0	1		0.5	1	
[23-TG]-C-3R	0.6	1		0.2	1		4.5	1	< 0.05
[24-TG]-A-4R	4.3	1	< 0.05	7.2	1	< 0.01	5.7	1	< 0.02
[24-TG]-C-4R	1.2	1		0.2	1		0.5	1	
[25-TG]-A-4R	4.4	1	< 0.05	5.3	1	< 0.03	0.5	1	
[25-TG]-A-3R		1		0.2	1		0.4	1	
[25-TG]-C-4R		1		0.2	1		0.4	1	
[26-TG]-A-4R	10.8	1	< 0.01	5.6	1	< 0.02	1.6	1	
[26-TG]-A-3R	4.3	1	< 0.05	4.6	1	< 0.05	0.1	1	
[28-TG]-A-3R	14.8	1	< 0.01	4.2	1	< 0.05	2.2	1	
[31-TG]-A-3R	6.5	1	< 0.02	4.6	1	< 0.05	1.8	1	
[31-TG]-C-3R	1.0	1		0.4	1		0.4	1	

Locus<br>Allele or haplotype	Frequency differences								
	17 sires (2) vs.<br>540 half sibs (3)			17 sires (2) vs.<br>540 dam haplotypes (4)			540 half sibs (3) vs.<br>540 dam haplotypes (4)		
	χ^2	d.f.	*P* - value	χ^2	d.f.	*P* - value	χ^2	d.f.	*P* - value
DelR242	0.3	1		10.9	1	< 0.01	72.6	1	< 0.01
nt-7(C>A)	0.05	1		1.0	1		14.8	1	< 0.01
nt456 (G>A)	1.9	1		9.7	1	< 0.01	29.8	1	< 0.01
5'UTR-(TG)n	6.2	9		21.9	9	< 0.01	50.1	9	< 0.01
19-TG[f]	0.3	1		0.6	1		2.7	1	
20-TG	0.5	1		2.3	1		9.1	1	< 0.01
21-TG	0.1	1		0.0	1		2.1	1	
22-TG	1.1	1		2.6	1		5.9	1	< 0.02
23-TG	0.0	1		0.6	1		6.1	1	< 0.02
24-TG	0.2	1		1.3	1		3.1	1	
25-TG	0.1	1		0.6	1		8.6	1	< 0.01

26-TG	0.5	1		0.9	1		3.6	1	
28-TG	3.4	1		15.3	1	< 0.01	26.0	1	<0.01
31-TG	0.1	1		0.2	1		0.7	1	
[*nt-7(C>A)*]-[*DelR242*]	3.2	3		13.3	3	< 0.01	41.8	3	< 0.01
A-4R	2.9	1		13.0	1	< 0.01	40.6	1	< 0.01
C-4R	0.2	1		0.7	1		1.7	1	
A-3R	1.9	1		6.6	1	< 0.02	11.1	1	< 0.01
C-3R	0.1	1		2.4	1		13.6	1	< 0.01
[*nt456(G>A)*]-[*DelR242*]	2.2	2		11.0	2	< 0.01	33.8	2	< 0.01
A-4R	0.2	1		0.5	1		2.1	1	
G-4R	1.9	1		9.7	1	< 0.01	29.8	1	< 0.01
A-3R	2.1	1		10.9	1	< 0.01	32.5	1	< 0.01
[*nt-7(C>A)*]-[*nt456(G>A)*]	2.9	3		13.6	3	< 0.01	33.8	3	< 0.01
A-A	1.7	1		8.1	1	< 0.01	18.5	1	< 0.01
C-A	0.1	1		0.7	1		3.2	1	
A-G	2.6	1		12.3	1	< 0.01	40.2	1	< 0.01
C-G	0.2	1		1.4	1		4.9	1	< 0.03
[*5'UTR-(TG)n*]-[*nt-7(C>A)*]-[*DelR242*]	18.4	20		40.0	20	< 0.01	131.9	20	< 0.001
[19-TG]-A-4R	0.8	1		1.0	1		2.8	1	
[19-TG]-A-3R	0.3	1		0.3	1		0.6	1	
[20-TG]-A-4R	1.3	1		3.5	1		12.3	1	< 0.01
[20-TG]-A-3R	0.3	1		0.3	1		0.6	1	
[20-TG]-C-4R	0.2	1		0.2	1		0.3	1	
[21-TG]-A-4R	0.8	1		1.0	1		2.8	1	
[22-TG]-A-4R	4.1	1	< 0.05	6.0	1	< 0.02	11.7	1	< 0.01
[22-TG]-A-3R	0.3	1		0.3	1		0.6	1	
[22-TG]-C-4R	0.1	1		0.4	1		1.4	1	
[23-TG]-A-3R	1.9	1		2.4	1		6.4	1	< 0.02
[23-TG]-C-4R	0.8	1		0.9	1		1.7	1	
[23-TG]-C-3R	0.2	1		3.7	1		27.6	1	< 0.01
[24-TG]-A-4R									

[24-TG]-C-4R	0.6	1		1.9	1		6.0	1	< 0.02
[25-TG]-A-4R	0.1	1		1.0	1		6.2	1	< 0.02
[25-TG]-A-3R	0.2	1		0.2	1		0.3	1	
[25-TG]-C-4R	0.2	1		0.2	1		0.3	1	
[26-TG]-A-4R	0.9	1		1.0	1		2.0	1	
[26-TG]-A-3R	0.2	1		1.0	1		9.2	1	< 0.01
[28-TG]-A-3R	7.1	1	< 0.01	19.2	1	< 0.01	50.9	1	< 0.01
[31-TG]-A-3R	0.4	1		0.5	1		0.9	1	
[31-TG]-C-3R	0.9	1		0.5	1		0.0	1	

[a]A total of 190 haplotypes derived from 95 sires.

[b]A total of 34 haplotypes derived from 17 sires of 540 half sibs. The sire genotypes were 14 *4R/3R* heterozygotes and three *3R/3R* homozygotes.

[c]A total number of 1080 haplotypes derived from 540 half sibs.

[d]A total number of 540 haplotypes derived from 540 dams of 540 half sibs. A dam's allele or haplotype was estimated by removing the sire's transmitted allele or haplotype from the half sib genotypes or haplotype combinations. The sire genotypes were 14 *4R/3R* heterozygotes and 3 *3R/3R* homozygotes.

[e]Degrees of freedom.

[f]$(TG)_{19}$.
