# Supplementary material for: Overdominance Effect of the Bovine Ghrelin Receptor (GHSR1a)-DelR242 Locus on Growth in Japanese Shorthorn Weaner Bulls: Heterozygote Advantage in Bull Selection and Molecular Mechanisms
Source: G3 (Bethesda). 2014 Dec 23;5(2):271–9. doi: 10.1534/g3.114.016105 (PMC4321035; doi:10.1534/g3.114.016105)
Supplement: Supporting Information [file supp_g3.114.016105_TableS3.pdf]

Table S3 Comparison of relative growth rates (RGR) of body shape and conformation traits among the *DelR242* genotypes in direct-tested weaner bulls.

Traits[a,b]	No. of animals	Mean (SD)[c] [%/month]	Genotype mean (SD) [%/month]			*P* - value[d]
			4R/4R	*4R/3R*	*3R/3R*	
BW[a]_RGR[b]	121	9.27	9.02[ab]	9.56[a]	8.90[b]	0.0085
		(0.85)	(0.84)	(0.83)	(0.80)	(*)
WH_RGR	121	2.11	2.05	2.11	2.21	0.3088
		(0.30)	(0.28)	(0.34)	(0.24)	(ns)
CW_RGR	94	4.13	3.16[c]	4.73[a]	3.96[ac]	0.0033
		(1.49)	(1.32)	(1.62)	(1.14)	(*)
RL_RGR	94	3.01	2.75	2.99	3.34	0.2306
		(0.98)	(0.90)	(1.04)	(0.93)	(ns)
CC_RGR	94	2.48	2.63	2.30	2.71	0.1528
		(0.75)	(0.85)	(0.63)	(0.80)	(ns)
No. of weaner bulls	121		33	61	27	
	94		25	47	22	

[a]Traits: BW, Body weight; WH, Withers height; CW, Chest width; CD, Chest depth; BL, Body length; RL, Rump length; HW, Hip width; TW, Thurl width; CG, Chest girth; CC, Cannon circumference in direct-tested weaner bulls

[b]Relative growth rate (RGR) [%/month] = {[(BMt2 - BMt1)/(t2 - t1)] / [(BMt2 + BMt1)/2]} × 100 × 30, where BMt1 or BMt2 denotes body measurement (BM) at the start of direct-testing (t1; days) or at the end of direct-testing (t2, days).

[c]SD, standard deviation.

[d]*P* - valued (Bonferroni correction), $P = 0.10/5 = 0.02$; $P = 0.05/5 = 0.01$; $P = 0.01/5 = 0.0025$. a,b: $P < 0.05/3 = 0.017$; a,c: $P < 0.01/3 = 0.003$.
