# Supplementary material for: Overdominance Effect of the Bovine Ghrelin Receptor (GHSR1a)-DelR242 Locus on Growth in Japanese Shorthorn Weaner Bulls: Heterozygote Advantage in Bull Selection and Molecular Mechanisms
Source: G3 (Bethesda). 2014 Dec 23;5(2):271–9. doi: 10.1534/g3.114.016105 (PMC4321035; doi:10.1534/g3.114.016105)
Supplement: Supporting Information [file supp_g3.114.016105_TableS4.pdf]

Table S4 Changes in the ratio of the *4R/3R* heterozygous individual in his progenies produced by mating between the progeny-tested sire and dams depending on the *3R* allele frequency in the dam population.

Allele frequency in the dam population		Genotype of the progeny-tested sire		
		4R/4R	*4R/3R*	*3R/3R*
4R	*3R*	Ratio of the *4R/3R* individual in his progenies (%)		
0.9	0.1	10	50	90
0.8	0.2	20	50	80
0.7	0.3	30	50	70
0.6	0.4	40	50	60
0.5	0.5	50	50	50
0.4	0.6	60	50	40
0.3	0.7	70	50	30
0.2	0.8	80	50	20
0.1	0.9	90	50	10
