# Supplementary material for: Overdominance Effect of the Bovine Ghrelin Receptor (GHSR1a)-DelR242 Locus on Growth in Japanese Shorthorn Weaner Bulls: Heterozygote Advantage in Bull Selection and Molecular Mechanisms
Source: G3 (Bethesda). 2014 Dec 23;5(2):271–9. doi: 10.1534/g3.114.016105 (PMC4321035; doi:10.1534/g3.114.016105)
Supplement: Supporting Information [file supp_g3.114.016105_TableS5.pdf]

Table S5 Summary statistics of growth, feed intake, body shape and conformation measurements traits in direct-tested bulls, carcass traits in shipped half-sibs, additive and dominance effects of the *C* allele of the *nt-7(C>A)* locus and epistatic effect between the *nt-7(C>A)* and *DelR242* loci.

Traits (abbreviation)[a](units)	No. of animals	Least squares mean						*P*-value[e]	Effect of the *C* allele				Epistatic effect[g]	
		A/A	(SD)[c]	*A/C*	(SD)	*C/C*	(SD)		LRT[f]	*P*-value	Addi-tive effect	Domi-nant effect	LRT	*P*-value
(1) Direct-tested bulls		(58)[d]		(41)		(22)								
AGE (day)	121	242.50	(20.7)	250.90	(20.5)	241.23	(19.2)	ns						
ADG (kg/day)	121	1.30	(0.14)	1.27	(0.15)	1.26	(0.15)	0.113[ns]	4.36	0.113	-0.03	-0.01	0.20	ns
180BW (kg)	121	244.40	(32.0)	235.60	(28.0)	248.70	(31.7)	0.562[ns]	1.153	0.562	1.53	-5.36	0.52	ns
365BW (kg)	121	486.7	(36.0)	470.5	(32.7)	482.9	(47.2)	0.228[ns]	2.959	0.228	-4.24	-8.29	0.04	ns
8SFT (mm)	121	79.7	(15.5)	81.5	(16.6)	77.7	(14.7)	0.023[ns]	7.56	0.023	0.00	5.24	0.39	ns
ROUGH (%)	121	45.0	(2.4)	44.8	(2.3)	45.2	(1.9)	0.776[ns]	0.51	0.776	0.15	-0.01	1.88	ns
ROUGH_1 (kg))	121	3.75	(0.48)	3.83	(0.52)	3.89	(0.48)	0.179[ns]	3.44	0.179	0.10	-0.02	0.09	ns
CONC_1 (kg)	121	4.50	(0.52)	4.61	(0.58)	4.63	(0.55)	0.360[ns]	2.04	0.360	0.08	-0.03	0.39	ns
Body shape and conformation measurements:														
WH_S (cm)	121	115.3	(3.4)	115.3	(3.5)	114.7	(3.9)	0.272[ns]	2.61	0.272	-0.49	-0.17	3.56	ns

WH_E (cm)	121	127.1	(3.1)	127.5	(3.4)	126.6	(3.7)	0.570^{ns}	1.17	0.570	-0.28	0.48	1.92	ns
		(40)		(34)		(20)								
CW_S (cm)	94	38.4	(3.4)	38.6	(4.0)	39.4	(4.0)	0.829^{ns}	0.37	0.829	0.07	-0.35	0.40	ns
CW_E (cm)	94	46.7	(2.8)	46.7	(3.2)	46.0	(2.7)	0.796^{ns}	0.46	0.796	-0.23	-0.08	0.81	ns
RL_S (cm)	94	42.7	(2.2)	42.8	(2.3)	41.9	(2.5)	0.496^{ns}	1.40	0.496	-0.28	0.01	0.05	ns
RL_E (cm)	94	48.8	(2.1)	48.9	(2.5)	49.3	(1.9)	0.412^{ns}	1.77	0.412	0.17	-0.50	1.05	ns
CC_S (cm)	94	17.7	(0.8)	17.6	(0.7)	17.6	(0.8)	0.576^{ns}	1.10	0.576	-0.11	-0.01	0.13	ns
CC_E (cm)	94	19.9	(0.6)	19.9	(0.7)	19.7	(0.9)	0.934^{ns}	0.14	0.934	-0.03	0.03	0.87	ns
(2) Shipped half sibs[b]		(282)		(208)		(47)								
Slaughter age (month)		24.2	(3.2)	24.0	(3.3)	24.6	(3.5)	—						
CW (kg)	537	405.0	(38.1)	409.6	(37.2)	407.0	(41.3)	0.282^{ns}	2.53	0.282	-0.57	4.97	0.83	ns
LMA (cm^2)	537	47.9	(5.2)	48.2	(5.3)	48.6	(5.4)	0.989^{ns}	0.02	0.989	0.06	0.00	0.54	ns
RT (cm)	537	6.37	(0.66)	6.39	(0.62)	6.38	(0.73)	0.646^{ns}	0.87	0.646	0.01	0.05	1.03	ns
SFT (cm)	537	2.35	(0.64)	2.43	(0.66)	2.42	(0.69)	0.093^{ns}	4.76	0.093	0.03	0.10	0.04	ns
BMS	537	2.11	(0.36)	2.12	(0.37)	2.04	(0.20)	0.422^{ns}	1.72	0.422	-0.02	0.05	0.01	ns
Firmness	537	2.09	(0.30)	2.09	(0.28)	2.04	(0.20)	0.512^{ns}	1.34	0.512	-0.03	0.02	0.11	ns

Texture	537	2.39	(0.50)	2.37	(0.48)	2.21	(0.41)	0.210^{ns}	3.12	0.210	-0.06	0.08	0.65	ns

[a]AGE, age at the start of direct-testing; ADG, average dairy gain; 180BW, 180-day adjusted body weight; 365BW, 365-day adjusted body weight; 8SFT, total thickness of eight points of subcutaneous fat; ROUGH, roughage intake; ROUGH_1, roughage weights per kilogram body weight gain; CONC_1, concentration weights per kilogram body weight gain; WH_S, withers height at the start of direct-testing; WH_E, withers height at the end of direct-testing; CW_S, chest width at the start of direct-testing; CW_E, chest width at the end of direct-testing; RL_S, rump length at the start of direct-testing; RL_E, rump length at the end of direct-testing; CC_S, cannon circumference at the start of direct-testing; CC_E, cannon circumference at the end of direct-testing; CW, carcass weight; LMA, longissimus muscle area; RT, rib thickness; SFT, subcutaneous fat thickness; BMS, beef marbling score.

[b]209 dams and 328 steers. [c]Standard deviation. [d]Number of animals. [e]*P* - value [(Significance level (Bonferroni correction): ns, not significant; **P* = 0.05/15 = 0.0033; 15, a number of traits for direct-tested bulls)]. [f]Likelihood ratio test. [g]Epistatic effect estimated by the epi_snp option between the *DelR242* and *nt-7(C>A)* loci .
